# Surveillance for severe acute respiratory infections (SARI) in hospitals in the WHO European region - an exploratory analysis of risk factors for a severe outcome in influenza-positive SARI cases

**DOI:** 10.1186/s12879-014-0722-x

**Published:** 2015-01-08

**Authors:** Tamara J Meerhoff, Artan Simaku, Dritan Ulqinaku, Liana Torosyan, Natalia Gribkova, Veronica Shimanovich, Giorgi Chakhunashvili, Irakli Karseladze, Aizhan Yesmagambetova, Ainagul Kuatbayeva, Zuridin Nurmatov, Dinagul Otorbaeva, Emilia Lupulescu, Odette Popovici, Elizaveta Smorodintseva, Anna Sominina, Olga Holubka, Olga Onyshchenko, Caroline S Brown, Diane Gross

**Affiliations:** Department of Primary and Community Care, Radboud University Medical Centre, Nijmegen, the Netherlands; Institute of Public Health, Tirana, Albania; State Hygiene and Anti-epidemic Inspectorate, Yerevan, Armenia; The Republican Research and Practical Center for Epidemiology and Microbiology (RRPCEM), Minsk, Belarus; The Republican Center for Hygiene, Epidemiology and Public Health, Minsk, Belarus; National Centre for Disease Control and Public Health (NCDC), Tbilisi, Georgia; Committee of Sanitary and Epidemiological Surveillance MOH, Astana, Kazakhstan; Scientifical-Practical Center of Sanitary and Epidemiological Expertise and Monitoring, Almaty, Kazakhstan; National Virological Laboratory, Department of the State Sanitary and Epidemiological Surveillance, Ministry of Health of Kyrgyz Republic, Bishkek, Kyrgyzstan; Department of State Sanitary Epidemiological Surveillance, Bishkek, Kyrgyzstan; Cantacuzino Institute, National Reference Centre for Influenza, Bucharest, Romania; National Institute of Public Health, National Centre for Communicable Diseases Surveillance and Control, Bucharest, Romania; National Influenza Centre at the Research Institute of Influenza, St Petersburg, Russian Federation; L.V. Gromashevskyi Institute of Epidemiology and Infectious Diseases, Kiev, Ukraine; WHO Regional Office for Europe, Copenhagen, Denmark

**Keywords:** Influenza, Europe, Risk factor, Severe acute respiratory infection, Surveillance

## Abstract

**Background:**

The 2009 H1N1 pandemic highlighted the need to routinely monitor severe influenza, which lead to the establishment of sentinel hospital-based surveillance of severe acute respiratory infections (SARI) in several countries in Europe. The objective of this study is to describe characteristics of SARI patients and to explore risk factors for a severe outcome in influenza-positive SARI patients.

**Methods:**

Data on hospitalised patients meeting a syndromic SARI case definition between 2009 and 2012 from nine countries in Eastern Europe (Albania, Armenia, Belarus, Georgia, Kazakhstan, Kyrgyzstan, Romania, Russian Federation and Ukraine) were included in this study. An exploratory analysis was performed to assess the association between risk factors and a severe (ICU, fatal) outcome in influenza-positive SARI patients using a multivariate logistic regression analysis.

**Results:**

Nine countries reported a total of 13,275 SARI patients. The majority of SARI patients reported in these countries were young children. A total of 12,673 SARI cases (95%) were tested for influenza virus and 3377 (27%) were laboratory confirmed. The majority of tested SARI cases were from Georgia, the Russian Federation and Ukraine and the least were from Kyrgyzstan. The proportion positive varied by country, season and age group, with a tendency to a higher proportion positive in the 15+ yrs age group in six of the countries. ICU admission and fatal outcome were most often recorded for influenza-positive SARI cases aged >15 yrs. An exploratory analysis using pooled data from influenza-positive SARI cases in three countries showed that age > 15 yrs, having lung, heart, kidney or liver disease, and being pregnant were independently associated with a fatal outcome.

**Conclusions:**

Countries in Eastern Europe have been able to collect data through routine monitoring of severe influenza and results on risk factors for a severe outcome in influenza-positive SARI cases have identified several risk groups. This is especially relevant in the light of an overall low vaccination uptake and antiviral use in Eastern Europe, since information on risk factors will help in targeting and prioritising vulnerable populations.

**Electronic supplementary material:**

The online version of this article (doi:10.1186/s12879-014-0722-x) contains supplementary material, which is available to authorized users.

## Background

Surveillance of influenza is important for determining the timing and spread of influenza, for tracking changes in circulating influenza viruses to inform seasonal influenza vaccine composition, and as an alert mechanism for potential pandemic viruses [1]. One of the gaps in influenza surveillance highlighted during the 2009 pandemic was the lack of systems that routinely monitor severe influenza. There was also limited timely information on risk factors associated with a severe outcome in hospitalised patients with influenza.

A recent evaluation of 2009 pandemic and seasonal influenza epidemics due to A(H1N1) and A(H3N2) viruses has shown that the morbidity by age group was similar, but that young age was an important risk factor for death only during the 2009 H1N1 pandemic [2]. In addition, obesity and pregnancy were identified as risk factors for a severe outcome of influenza A(H1N1)pdm09 infection compared to seasonal influenza [3] and having an underlying disease was a risk factor for a severe outcome due to pandemic and seasonal influenza viruses [4-7]. However, most of the studies on risk factors for a severe outcome of influenza that are available are from developed and/or high income countries; there is limited information on risk groups in low- and middle-income countries [8,9] and the role of influenza in SARI patients for countries in Central and Eastern Europe, where the antiviral treatment and vaccination uptake [10,11] are low.

Sentinel surveillance for hospitalised patients meeting a syndromic Severe Acute Respiratory Infection (SARI) case definition has recently been established in countries in the World Health Organization (WHO) European Region. By the end of the first post-pandemic influenza season (2010–2011), a total of 11 countries had established sentinel SARI surveillance using standard methods based on guidance published by the WHO Regional Office for Europe [12]. This paper describes the characteristics of SARI patients and investigates risk factors for a severe outcome (ICU/fatal) in influenza-positive SARI patients in countries in Central and Eastern Europe.

## Methods

### Data collection

Countries that performed influenza surveillance in the WHO European Region and collected case-based data on SARI patients as part of their national influenza surveillance between 2009 and 2012 were invited to participate in the study. For inclusion, as a minimum, data on age, gender, clinical symptoms, presence and specification of underlying conditions, and influenza (sub)type laboratory test results were required for each case. Testing of all hospitalised patients that met the SARI case definition at the sentinel sites was recommended in the WHO Euro guidelines [12]. Sampling and testing procedures were the same during and after the pandemic.

Countries that met the inclusion criteria on the minimum data requirements were asked to provide the following information on SARI patients: geographical region/district of the hospital, hospital, gender (male, female), date of birth/age, case definition, date onset influenza, sampling date, sample type (nasal swab, throat swab, combined nasal/throat swab, aspirate, bronchoalveolar lavage (BAL), tissue from biopsy or autopsy, other, unknown/not specified), clinical symptoms, existing underlying conditions (yes/no), asthma (yes/no), diabetes (yes/no), cancer (yes/no), immune-compromised (yes/no), heart (yes/no), kidney (yes/no), lung (yes/no), liver (yes/no), neurological (yes/no), obesity (no, BMI 30–40, BMI > 40, clinically obese), pregnancy (yes/no), pregnancy trimester (1^st^ trimester, 2^nd^ trimester, 3^rd^ trimester), vaccination status (yes/no), antiviral prophylaxis (yes/no), antiviral treatment (yes/no), antiviral resistance (yes/no), respiratory support (no, oxygen, ventilation, ECMO), pneumonia diagnosis (no, yes (clinical/abnormal chest X-ray/raised CRP level), Tuberculosis (TB) (no history of TB, history of TB, positive test during hospital), influenza test (not performed, polymerase chain reaction (PCR), culture, immunofluorescent assay (IFA), other -not specified- test), test result (negative/positive), influenza type (A/B), influenza A subtype (A(H1N1)pdm09, A(H3N2)), ICU admission, outcome (discharged alive/death), and cause of death (not influenza, influenza as a primary cause, influenza as a secondary cause).

A descriptive analysis was performed to investigate the SARI patient characteristics by influenza status and country. The season was defined from 1 August to 31 July the following year. Pregnancy and obesity were included when determining the total number of multiple underlying conditions. Except for pregnancy, the percentage of patients with a specific condition was calculated by dividing the total number of patients with the condition by the total number of patients with available data for that variable. The percentage of pregnant women was defined by dividing the number of pregnant women by the number of women of childbearing age (15–49 yrs).

### Statistical analysis

We assessed the association between possible risk factors (i.e. age (categorical), gender, underlying conditions, influenza subtype) and different levels of severity for influenza-positive SARI patients. Only cases with available risk data were included in the analysis. Four patient outcomes were defined: 1) SARI patients that were not admitted to ICU and discharged alive, 2) SARI patients admitted to ICU and discharged alive, 3) SARI patients that died, and 4) SARI patients with any severe outcome (patients that were admitted to ICU or died). The three more severe levels were compared to the least severe outcome: SARI patients who were not admitted to ICU and discharged alive. The influenza subtype A(H1N1)pdm09 (vs. non-influenza subtype A(H1N1)pdm09) was also included as possible risk factor for a severe outcome. Because data on vaccination status and antiviral treatment were incomplete -or numbers were very low- we did not include these variables in the data analysis.

Logistic regression analysis was performed to identify factors associated with a severe outcome. All variables associated with a severe outcome at a significance level of p <0.15 were included in a multivariate logistic regression analysis (Enter method) to identify factors independently associated with ICU admission or death. A factor was defined significant when p < 0.05 in the multivariate analysis. For categorical variables with >2 levels, the Chi-squared test was used, while for categorical variables with 2 categories (2×2 table) the Continuity Correction was used. Correlation between variables was checked and if the Pearson correlation was >0.5, we included only one of the variables in the analysis. Finally, a pooled data analysis was performed for countries that had collected data on both ICU admission and deaths. Variables that were significant in the univariate logistic regression analysis were included in the multivariate analysis. To control for differences between countries, the country (Romania = reference category, Albania = dummy1, Georgia = dummy2) was included as a factor in the model. SPSS 20 was used for the analyses.

### Ethical considerations

Verbal consent was obtained from all patients before specimen collection as per country’s routine public health practice. WHO Regional Office for Europe considered that anonymised data collected through sentinel hospital surveillance for influenza to be part of routine public health surveillance; therefore, formal ethical review was not required.

## Results

Eleven countries performed SARI surveillance and were contacted to participate in the study. Nine countries (Albania, Armenia, Belarus, Georgia, Kazakhstan, Kyrgyzstan, Romania, Russian Federation and Ukraine) fulfilled the inclusion criteria and were included in the study. For each country a description of the population, age distribution and other health-related topics is available in Table 1 [13]. The population of the countries participating in this project varies from 3.2 million in Armenia and Albania to 142.6 million in the Russian Federation. The age distribution is similar for the countries with the exception of Kazakhstan and Kyrgyzstan where children (0–14) represent a substantial (24-30%) proportion of the population. The number of hospitals available per 100,000 of the population range from 1.4 in Albania to 7.0 in Belarus. Outpatient contacts per person -the total number of primary health care or ambulatory care contacts divided by the population- vary by country. They are relatively low at in Albania (2.0) and Georgia (2.1), and high in Belarus (13.2) and Ukraine (10.7). The system characteristics including SARI case definitions are presented in Table 2 and additional information on the SARI surveillance descriptions can be found at the WHO Regional Office for Europe website [14].

**Table 1:** **Country population and health care data**

	**Albania**	**Armenia**	**Belarus**	**Georgia**	**Kazakhstan**	**Kyrgyzstan**	**Romania**	**Russian Federation**	**Ukraine**
**Mid-year population** (in million)	3.2	3.2	9.5	4.5	16.4	5.5	21.4	142.6	45.6
**% population aged** 0**–**14 years	-	18.5	17.0	17.0	24.4	30.2	15.1	15.2	14.2
**% population aged** 65+ years	-	10.3	13.9	13.8	6.7	4.5	14.9	12.8	15.5
**Live births/year** (x1000) *(2009)*	34	44.4	109.2	63.3	357.5	135.5	222.4	1761.7	512.5
**Crude death rate per 1000 pop** *(2009)*	-	8.5	13.97	10.57	8.97	6.67	11.98	14.17	15.41
**Hospitals per 100.000 population**	1.4	4.0	7.0	6.2	6.1	2.7	2.6	na	6.1
**Hospital beds per 100.000 population**	267	381	1126	286	725	476	634	na	919
**Outpatient contacts/person per year**	2.0	3.5	13.2	2.1	6.9	3.6	4.7	9.5	10.7

Source: European Health for All database (HFA-DB) (http://data.euro.who.int/hfadb/). The mean for the period 2009–2012 was calculated, except for % population where data from 2009/2010 are presented.

**Table 2:** **SARI data collection in nine countries in the WHO European Region, 2009-2012**

	**Albania**	**Armenia**	**Belarus**	**Georgia**	**Kazakhstan**	**Kyrgyzstan**	**Romania**	**Russian Federation**	**Ukraine**
**Number of sentinel hospitals**	15	6/7	11	6	17	4	12-26	19	9-10
**Period data collection**	Nov 2009- Mar 2011	Dec 2010-Mar 2012	Sep 2010-Dec 2012	Jan 2009-Mar 2012	Sep 2011-Dec 2012	Nov 2010-Jun 2011	Oct 2009-May 2012	Sep 2010-Dec 2012	Sep 2009-Dec 2012
**Case definition -all ages**			Standard^a^	New^cd^	Standard^ae^		New^cd^		
*Case definition <5*	WHO^b^	Pneum^b^				Pneum^b^	Pneum^b^	Pneum^b^	Pneum^b^
*Case definition ≥ 5*	Standard^a^	Standard^a^				Standard^a^	Standard^a^	Standard^a^	Standard^a^
**No. SARI cases**	102	335	1025	2138	857	369	1003	2779	4667
No. children aged 0–14 (%)	24 (23.5%)	287 (85.7%)	412 (40.1%)	1137 (53.5%)	538 (62.8%)	267 (72.4%)	491 (49.0%)	1718 (61.8%)	2339 (50.1%)
No. SARI tested influenza (%)	102 (100%)	188 (56.1%)	1025 (100%)	2138 (100%)	834 (97.3%)	43 (11.6%)	914 (91.1%)	2779 (100%)	4650 (99.6%)
**Type of hospital included**	ID, PED, PULM, ICU	GEN, ID, PED, EM, GYN	GEN, ID, PED, EM	GEN, ID, PED, EM, ICU	ID, PED, EM	GEN, ID, PED	ID, PED, PULM, EM	GEN, ID, PED, EM	GEN, ID, PED
**Number of beds per hospital**	76-436	50-500	1000	63-250	100-395	180-400	92-1556	na	110-645
**Catchment area and/or site selection**	1 hospital in each of 12 counties + 3 hospitals in Tirana.	1 hospital in 2 regions and 5 hospitals in Yerevan.	The 11 hospitals are located in large cities of the country’s 6 regions.	SARI admission rates and collaboration NCDC used to identify sites^e^ Most regions of the country were covered.	The hospitals located in 7 regions of the country.	Sites are located in 2 cities (Bishkek and Osh). The sites serve about 1.5 million of the population.	Sites are regional clinical hospitals, well equipped for care to SARI cases, and cover 19-30% of population.	Sites (1–2) are located in 9 cities in 6 Federal districts. The 9 cities cover a population of 10.5 million.	Sites (10) are located in 4 cities in different geographical parts of the country.
**Monitoring data quality**	Sentinel site visit	Sentinel site visit. Monthly during season	Weekly basis	Monthly during season^f^	All year round	Two times a year at the national level	Weekly at the national level by the SARI coordinator.	Weekly basis	Weekly at the national level by the SARI coordinator.
**Vaccination recommendations (rec)** ^**g**^ **/risk groups and coverage**	Children < 5, Elderly >65, persons with underlying diseases, HCW	WHO rec.	WHO rec. Pregnant women obligatory since 2011	WHO rec. Pregnant women, persons with underlying diseases^h^, > 65 yrs, children <2. Coverage in risk groups is 90%	WHO rec. Coverage in risk groups is 98-100%	Vaccination coverage in risk groups is about 3% (HCW, children and pilgrims)	WHO rec. Persons 6 months-64 years old with chronic underlying conditions, pregnant women, HCW, staff working with institutionalised persons, residents of the social care institutions, persons ≥65 years old. Coverage is: Tot: 5.2-14.6%; 65+: 19.1%-49.4% HCW: 51%-97.8%	WHO rec. >30 mln of population each year	Age >60, underlying disease (chronic cardio-vascular, lung, kidney, liver, HIV, diabetes, primary immunodeficiency), pregnancy, HCW
**Method influenza detection** ^**i**^	PCR	PCR, culture	PCR	PCR	PCR, other, culture	PCR, other	Real-time PCR for type and subtype detection	PCR	PCR, other

*Abbreviations: ICU* Intensive care unit, *Na* not available, *PCR* polymerase chain reaction, *SARI* severe acute respiratory infection, *GEN* general/multi-profile/national referral, *ID* infectious disease, *PED* paediatrics, *EM* emergency, *PULM* Pulmonology, *GYN* obstetrics and gynecology/maternal department, *HCW* Health care workers.

^a^The standard SARI case definition is defined as a patient with onset of the following symptoms ≤ 7 days prior to hospitalisation:

Fever >38°C AND cough OR sore throat AND shortness of breath or difficulty in breathing.

^b^The WHO case definition is defined for pneumonia and severe pneumonia in children below the age of 5, and is as follows:

Pneumonia: cough OR difficulty breathing AND breathing faster than 40 breaths/minute (12–59 month) or breathing faster than 50 breaths/minute (2–11 month);

Severe pneumonia: cough OR difficulty breathing AND any of the following severe signs: unable to drink or breastfeed, OR vomits everything, OR convulsions, OR lethargic or unconscious, OR chest indrawing or stridor in a calm child.

^c^New WHO case definition – all ages.

An acute respiratory illness with onset in the 7 days prior to hospital admission, that results in hospitalization over night and includes:

History of fever or measured fever of ≥ 38°C, AND cough, AND shortness of breath or difficulty breathing.

^d^The new WHO case definition cd^c^ was used in the 2011–2012 influenza season, for the seasons before 2011–2012 the WHO case definition^ab^ was used.

^e^Sites were selected according to their SARI admission rates and collaboration with NCDC, most regions of the country were covered. In the first year 2008–2009 the surveillance was nationwide, in 2009–2010 a transition to a sentinel system was made, and was capable to provide representative data *(personal communication)*.

^f^The sentinel epidemiologist monitors the data collection at a regular basis. The NCDC specialist visits the sentinel site and checks sentinel data on quarterly basis and monthly during the active influenza season *(Personal communication Giorgi Chakhunashvili).*

^g^Recommended risk groups for seasonal influenza vaccination are: pregnant women (highest priority) and in no particular order of priority: children aged 6 to 59 months, the elderly, individuals with specific chronic medical conditions, and health-care workers [8].

^h^Cardiovascular disorder, respiratory disorder, kidney disorders, hepatitis, HIV, diabetes, immunocompromised persons, oncological patients.

^i^In case culture is used, this is performed in 1% of all tests.

Note: country-specific information in this table (type of hospital included, number of beds per hospital, catchment area and/or site selection, monitoring data quality, vaccination recommendations) are kindly provided by the co-authors of this paper (personal communication).

From 2009 to 2012, a total of 13,275 SARI patients were reported. Overall, the majority of SARI patients reported in these countries were young children and the large majority of SARI patients (95%) were tested for influenza virus. This small group of SARI patients that were not tested were generally children with no underlying medical conditions (Additional file 1: Table A). Different types of hospitals were included in the SARI surveillance (Table 2), of which infectious disease hospitals, general/multi-profile/national referral hospitals, paediatric hospitals and emergency hospitals were more common. In Albania and Georgia ICU facilities were included in the surveillance and in Armenia an obstetrics and gynaecology/maternity department was included. The number of beds per hospital and other surveillance-related topics such as the recommended risk groups for vaccination [8] can be found in Table 2. An assessment on the proportion of SARI cases admitted and cases included in the surveillance were only available for Romania and amounted 25% of SARI patients *(Personal communication: Odette Nicolae)*.

The results of SARI surveillance varied by country and the number of SARI samples tested, samples tested positive for influenza, the virus detections and fatal cases are summarised by country, season and age group in Table 3. Georgia reported data for four influenza seasons (from start of 2009 till 2012), while Kazakhstan (2011–2012) and Kyrgyzstan (2010–2011) collected data for one influenza season. Overall, the highest proportion of influenza-positive cases were reported in Albania (100% in 2009–2010) and in Georgia (75.2% in 2010–2011). The proportion positive varied by country, season and age group, with a tendency to a higher proportion positive in the 15+ yrs age group in Albania, Armenia, Kazakhstan, Romania, the Russian Federation and Ukraine. The highest proportion of pregnant women was observed in influenza-positive SARI patients in Armenia (73%) and the Russian Federation (61%), and lowest in Kyrgyzstan (0%), Albania (12.5%) and Ukraine (15%). See Additional file 1: Table A.

**Table 3:** **Description of SARI patients testing positive for influenza by country, season and age group (<15, 15+)**

	**Country and season**	**Samples tested**	**Samples positive (%)**	**Influenza A**	***Influenza A(H1N1) pdm09***	***Influenza A(H3N2)***	***Influenza A (not sub- typed)***	**Influenza B**	**Fatal cases (%)**
**Albania**	**2009-2010**	**58**	**58 (100%)**	**58**	***55***	***3***	***0***	**0**	**13/58 (22.4%)**
	<15 years	12	12 (100%)	12	*11*	*1*	*-*	-	0/12 (0%)
	15+ years	46	46 (100%)	46	*44*	*2*	*-*	-	13/46 (28.3%)
	**2010-2011**	**44**	**10 (22.7%)**	**10**	***8***	***2***	***0***	**0**	**0/10 (0%)**
	<15 years	12	2 (16.7%)	2	*2*	*0*	*-*	-	-
	15+ years	32	8 (25%)	8	*6*	*2*	*-*	-	-
**Armenia**	**2010-2011**	**140**	**22 (15.7%)**	**17** ^**a**^	***9***	***0***	**-**	**5**	**1/22 (4.5%)**
	<15 years	109	8 (7.3%)	5^a^	*0*	*0*	*-*	3	1/8 (12.5%)
	15+ years	31	14 (45.2%)	12^a^	*9*	*0*	*-*	2	0/22 (0%)
	**2011-2012**	**48**	**1 (2.1%)**	**1**	***0***	***1***	**-**	**0**	**0/1**
	<15 years	39	0 (0%)	0	*-*	*0*	*-*	0	-
	15+ years	9	1 (11.1%)	1	*-*	*1*	*-*	0	0/1
**Belarus**	**2010-2011**	**380**	**35 (9.2%)**	**24**	***24***	**-**	**-**	**11**	**Na**
	<15 years	105	10 (9.5%)	7	*7*	*-*	*-*	3	
	15+ years	275	25 (9.1%)	17	*17*	*-*	*-*	8	
	**2011-2012**	**635**	**16 (2.5%)**	**16**	***1***	***15***	**-**	**0**	**Na**
	<15 years	306	8 (2.6%)	8	*1*	*7*	*-*	-	
	15+ years	329	8 (2.3%)	8	*0*	*8*	*-*	-	
**Georgia**	**2008-2009**	**196**	**46 (23.5%)**	**42**	***39***	***3***	***0***	**4**	**0/46 (0%)**
	<15 years	163	33 (20.2%)	29	*26*	*3*	*-*	4	0/33 (0%)
	15+ years	33	13 (39.4%)	13	*13*	*0*	*-*	0	0/13 (0%)
	**2009-2010**	**1526**	**531 (34.8%)**	**499**	***484***	***11***	***4***	**32**	**31/531 (5.8%)**
	<15 years	1051	327 (31%)	296	*281*	*11*	*4*	31	7/327 (2.1%)
	15+ years	475	204 (2.3%)	203	*203*	*0*	*0*	1	24/204 (11.8%)
	**2010-2011**	**375**	**282 (75.2%)**	**146**	***143***	***1***	***2***	**136**	**53/282 (18.8%)**
	<15 years	196	150 (76.5%)	63	*62*	*1*	*0*	87	3/150 (2.0%)
	15+ years	179	132 (73.7%)	83	*81*	*0*	*2*	49	50/132 (37.9%)
	**2011-2012**	**21**	**10 (47.6%)**	**10**	***5***	***5***	***0***	**0**	**7/10 (70%)**
	<15 years	6	4 (66.7%)	4	*4*	*0*	*-*	-	1/4 (25%)
	15+ years	15	6 (40%)	6	*1*	*5*	*-*	-	6/6 (100%)
**Kazakhstan**	**2011-2012**	**791**	**186** ^**a**^ **(23.5%)**	**171** ^**a**^	***54***	***100***	***2***	**10**	0/38*
	<15 years	509	95^a^ (18.7%)	90^a^	*23*	*59*	*-*	2	0/21*
	15+ years	282	91^a^ (32.3%)	81^a^	*31*	*41*	*2*	8	0/17*
**Kyrgyzstan**	**2010-2011**	**43**	**19 (44.2%)**	**19**	***10***	***9***	**-**	**-**	**Na**
	<15 years	13	13 (100%)	13	*5*	*8*			
	15+ years	30	6 (20%)	6	*5*	*1*			
**Romania**	**2009-2010**	**211**	**66 (31.3%)**	**66**	***66***	***0***	**-**	**0**	**11/66 (16.7%)**
	<15 years	75	13 (17.3%)	13	*13*	*0*	*-*	-	2/13 (15.4%)
	15+ years	136	53 (39%)	53	*53*	*0*	*-*	-	9/53 (17%)
	**2010-2011**	**422**	**165 (39.1%)**	**84**	***83***	***1***	**-**	**81**	**21/165 (12.7%)**
	<15 years	203	69 (34%)	18	*18*	*0*	*-*	51	0/69 (0%)
	15+ years	219	96 (43.8%)	66	*65*	*1*	*-*	30	21/96 (21.9%)
	**2011-2012**	**281**	**69 (24.6%)**	**68**	***0***	***68***	**-**	**1**	**1/69 (1.4%)**
	<15 years	173	31 (17.9%)	31	*0*	*31*	*-*	0	1/31 (3.2%)
	15+ years	108	38 (35.2%)	37	*0*	*37*	*-*	1	0/38 (0%)
**Rus. Fed.**	**2010-2011**	**1293**	**274 (21.2%)**	**203** ^**a**^	***178***	***22***	**-**	**71**	**Na**
	<15 years	834	103 (12.4%)	73^a^	*62*	*10*	*-*	30	
	15+ years	459	171 (37.3%)	130^a^	*116*	*12*	*-*	41	
	**2011-2012**	**1486**	**153** ^**a**^ **(10.3%)**	**128**	***8***	***120***	**-**	**23**	**Na**
	<15 years	884	49 (5.5%)	42	*2*	*40*	*-*	7	
	15+ years	602	104^a^ (17.3%)	86	*6*	*80*	*-*	16	
**Ukraine**	**2009-2010**	**2149**	**731 (34%)**	**672**	***666***	***0***	***6***	**59**	**10/730 (1.4%)**
	<15 years	954	248 (26.0%)	231	*229*	*0*	*2*	17	0/247 (0%)
	15+ years	1195	483 (40.4%)	441	*437*	*-*	*4*	42	10/483 (2.1%)
	**2010-2011**	**2046**	**490 (23.9%)**	**219** ^**a**^	***200***	***5***	***13***	**271**	**0/489 (0%)**
	<15 years	1134	208 (18.3%)	105	*101*	*1*	*3*	103	0/208
	15+ years	912	282 (30.9%)	114^a^	*99*	*4*	*10*	168	0/281
	**2011-2012**	**413**	**188** ^**a**^ **(45.5%)**	**179** ^**a**^	***0***	***171***	***1***	**6**	**Na**
	<15 years	251	107^a^ (42.6%)	102^a^	*0*	*94*	*0*	2	
	15+ years	162	81 (50%)	77	*0*	*77*	*0*	4	

Na: not available; *Incomplete data; ^a^The influenza (sub)type information were not available for all influenza-positive SARI patients.

Note: for the countries Albania, Belarus, Georgia and Ukraine the total number of samples tested and/or tested positive are slightly lower compared to the data presented in the Supplement, this is due to the selection and availability of the influenza type and subtype data, and season/age group information.

**Figure 1:**
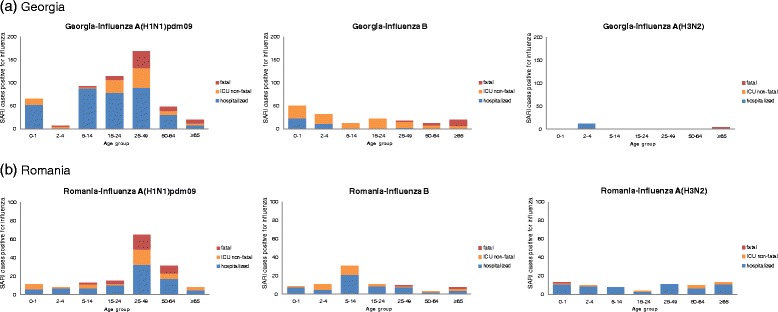
**SARI patients presented by age group and influenza (sub)type in two countries, 2009–2012.**

The (sub)typed influenza viruses reported differed by season, e.g. in Georgia in the 2010–2011 season influenza B (48.2%) co-circulated with influenza A(H1N1)pdm09 (50.7%), and a substantial proportion of fatal cases in the older adults were infected with influenza B (Figure 1). In Ukraine the influenza A(H1N1)pdm09 virus was dominant (91%) in the 2009–2010 influenza season. Influenza A(H1N1)pdm09 (40.8%) and B (55.3%) were co-dominant in the 2010–2011 season and influenza A(H3N2) was dominant (91%) during the 2011–2012 influenza season. A similar pattern could be observed for Romania (Table 3). In terms of fatal cases these were most often recorded in patients aged >15 yrs of age.

Data on the ICU admission status of hospitalised SARI patients were collected by six countries (Armenia, Albania, Belarus, Georgia, Kazakhstan and Romania). ICU admission in influenza-positive SARI patients ranged from 6.3% in Kazakhstan to 56.5% in Armenia. Information on deaths was available for Albania, Armenia, Georgia, Romania and Ukraine; the % of influenza-positive SARI patients that died ranged from 0.8% in Ukraine to 18.8% in Albania. See for detailed characteristics by country the Additional file 1: Table A.

**Figure 2:**
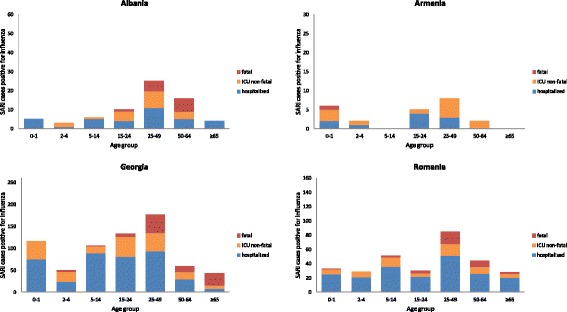
**SARI patients positive for influenza presented by age group in four countries, 2009–2012.**

In Figure 2 the influenza-positive SARI cases are presented by severity; cases not admitted to ICU, non-fatal cases admitted to ICU, and cases with a fatal outcome. Data on both ICU and fatal cases were available for Albania, Armenia, Georgia and Romania and are presented. Overall, most fatal SARI cases occurred in the age groups 25–49 yrs and 50–64 yrs with only one fatality reported in the 0–2 year old age group, but this was identified as being “not due to influenza”. In Georgia, a substantial number of fatal cases were reported in the age group >65 yrs. We also evaluated the detected influenza virus (sub)type and severity for Georgia and Romania, as they had sufficient data for this (Figure 1). Most SARI patients were influenza A(H1N1)pdm09 positive. Data from Georgia presenting influenza B positive patients indicated that practically all adults were admitted to ICU or had a fatal outcome. A relatively low number of SARI patients tested positive for influenza A(H3N2) in the period 2009–2012.

Influenza vaccination rates in all countries were very low. Seven out of nine countries had data on the vaccination status of the SARI cases. For the influenza-negative SARI patients the proportion vaccinated for influenza ranged from 0–3.2%, and for influenza-positive cases 0–4.1%. The use of antiviral medication to treat influenza-positive SARI patients was low and ranged from 0.5% in Kazakhstan to about 35% in Armenia (Additional file 1: Table A). Generally, the neuraminidase inhibitor oseltamivir was used as antiviral treatment.

To explore a possible association between known risk factors and a severe outcome, we presented the risk factors for influenza-positive SARI patients by outcome: patients that were not admitted to ICU, patients admitted to ICU, patients that died and patients that were admitted to ICU or who died (Additional file 1: Table B). We observed that a substantial proportion of the patients admitted to the ICU or that died had underlying medical conditions. The univariate logistic regression analysis on the country level revealed that age >15 yrs, being infected with influenza A(H1N1)pdm09, and having underlying conditions (in particular being immune-compromised, or pregnant or having lung or heart disease) were significantly associated with a fatal outcome (Additional file 1: Table C).

**Figure 3:**
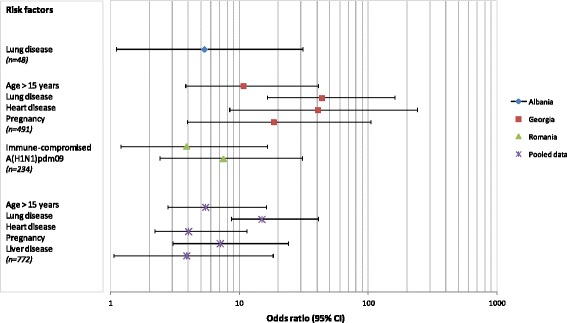
**Effect of risk factors on a fatal outcome in influenza-positive SARI patients.**

In the multivariate analysis using logistic regression we investigated the association of previously identified risk factors in influenza-positive SARI patients with a severe outcome (i.e. being fatal, admitted to ICU or fatal/ICU) for Albania, Georgia and Romania (Figure 3). We excluded data from Armenia from the multivariate analysis because of insufficient data. The data were not representative for the general population, as evidenced by the extremely high proportion of pregnant women in the data. Below we present the results for the logistic regression analyses by country (Albania, Georgia and Romania) followed by the pooled data analysis results.

The individual country results indicate that for Albania influenza-positive SARI patients with lung disease were at increased risk for a fatal outcome (OR = 5.33, CI95% = 1.10-25.76), and fatal outcome/ICU admission (OR = 5.42, CI 95% = 1.29-22.69) (Additional file 1: Table 1D).

In Georgia the following variables were independent risk factors for a fatal outcome in influenza-positive SARI patients: age > 15 yrs (OR = 10.73, CI 95% = 3.80-30.28), lung disease (OR = 43.65, CI 95% = 16.46-115.7), heart disease (OR = 40.62; CI 95% = 8.34-198.0) and being pregnant (OR = 18.39, CI 95% = 3.92-86.38). For ICU admission lung disease (OR = 5.94, CI 95% = 3.35-10.58) and heart disease (OR = 10.47; CI 95% = 3.45-31.83), as well as being pregnant (OR = 6.02, CI 95% = 2.22-16.33) were independent risk factors for ICU admission in influenza-positive SARI patients. Age > 15 yrs (OR = 9.75; CI 95% = 4.61-20.61), having lung disease (OR = 14.90, CI 95% = 8.14-27.27), kidney disease (OR = 8.78, CI 95% = 1.50-51.29) and being pregnant (OR = 3.27, CI 95% = 1.13-9.49) were independent risk factors for influenza-positive SARI patients admitted to ICU or with a fatal outcome (Additional file 1: Table 4D).

In Romania the following variables were independent risk factors for a fatal outcome in influenza-positive SARI patients: being immune-compromised (OR = 3.87, CI 95% = 1.19-12.60) and being infected with influenza A(H1N1)pdm09 virus (OR = 7.48, CI 95% = 2.39-23.36). In Romania, the influenza subtype A(H1N1)pdm09 was also an independent risk factor (OR = 2.40, CI 95% = 1.39-4.12) for patients with any severe outcome (admitted to ICU or with a fatal) (Additional file 1: Table 7D).

For the pooled data analysis the following variables were independent risk factors for a fatal outcome in influenza-positive SARI patients when controlling for the effect of country: age > 15 (OR = 5.44, CI 95% = 2.77-10.71), lung disease (OR = 14.89, CI95% = 8.62-25.71), heart disease (OR = 4.01, CI95% = 2.21-7.29), liver disease (OR = 3.59, CI95% = 1.11-11.42), kidney disease (OR = 3.88, CI95% = 1.06-14.28) and pregnancy (OR = 7.08, CI95% = 3.01-16.68), see Figure 3. Although obesity was a significant risk factor for severe outcome in some countries in the univariate logistic regression analysis, we were not able to include this in the pooled analysis due to incomplete data. No results are presented for ICU patients and fatal/ICU patients, as the Hosmer and Lemeshow test for these outcomes were significant (p = 0.000) and indicated a bad fit with the model.

## Discussion

Severe influenza surveillance has been established in nine countries in Eastern Europe and an exploratory analysis on risk factors for a severe outcome has been performed for data from Armenia, Georgia and Romania. We observed that most SARI patients admitted to the hospitals were young children and that influenza-positive SARI patients were generally more often admitted to ICU and resulted more often in death than the influenza-negative SARI patients. Influenza A(H1N1)pdm09 was generally detected in the 2009–2010 season, whereas influenza B and influenza A(H1N1)pdm09 were most frequently observed in the 2010–2011 season with influenza B being most prominent in children. Influenza A(H3N2) was commonly detected in the 2011–2012 season. Overall, our results indicated that ICU admission rates for influenza-positive SARI cases ranged from 6-56%, and fatal cases from 0.8-18.8%. These findings differed by country, season and age group. The proportion of ICU admissions and fatal cases is similar to what has been reported in SARI cases in nine EU countries by Snacken et al. [15] where 37% of hospitalised cases were admitted to ICU, and 15.6% died, and a study from Bagdure et al. [16] where 26% of hospitalised children infected with influenza A(H1N1)pdm09 were admitted to the ICU and 3% died.

Pooled data analysis identified risk factors (>15 yrs of age, having lung, heart, kidney or liver disease or being pregnant) for a fatal outcome in influenza-positive SARI patients. Risk factors for severe outcome for influenza-positive SARI patients did differ slightly between countries, but overall corresponded to risk factors for a severe outcome that have been reported in the literature worldwide [7,17] and were similar to findings in Spain [18]. For Western Europe limited data are available on hospitalised severe cases [6,15]. With most of the studies in the literature focusing on risk factors for a severe outcome in cases with influenza A(H1N1)pdm09 during the pandemic, our study evaluated both pandemic and seasonal viruses from 2009–2012. Therefore we need to be careful in interpreting the results. While literature has shown that the main risk groups such as having underlying disease are similar for influenza A(H1N1)pdm09 and seasonal influenza, obesity was identified as new risk factor for a severe outcome [7]. Many studies, however, have a lack of power and more evidence is needed to improve the level of evidence to identify risk factors for a severe outcome for pandemic and seasonal influenza [19].

The risk factors for a severe outcome (being > 15 yrs of age and having underlying disease) were similar for the three countries but there were also some country differences. Data from Romania indicated that being immune-compromised and being infected with the influenza A(H1N1)pdm09 virus were risk factors for a fatal outcome, but this effect was not observed in the pooled data analysis. Data for Georgia showed that many patients in ICU and/or fatal cases were influenza B positive and this is different from Romania. These findings may represent country differences, but could also be due to differences in surveillance systems, local practices or differences in circulating viruses. Unfortunately, we were not able to further investigate the role of the different virus (sub)types on the outcome due to limited data, including differences in the seasonal and pandemic influenza epidemiology.

One of the strengths in this study is the use of a standard case definition and collecting information on both influenza-negative and positive SARI cases. Admission criteria for the hospital and treatment are generally based on the judgement of the clinician and hospital admissions may not always be related to severity. Therefore using a case definition is important. Although a common approach was used, we observed country differences in the proportion of children, pregnant women and elderly being admitted to the hospital. These differences may be partly explained by a different population structure, probability of seeking care [20], but also by the inclusion of different hospital types and wards in the surveillance system - e.g. the high proportion of pregnant women in Armenia may be due to the inclusion of the obstetrics/maternity department. Furthermore, young children with a respiratory infection may be more likely to be admitted to the hospital than adults and this may indicate that different case management policies and criteria are used for hospital admission.

The proportion of SARI patients testing positive varied between countries and should be interpreted with caution. For Albania (2009–2010), Georgia (2010–2011) and Kyrgyzstan (2010–2011, age group <15 yrs) the positivity rates were unexpectedly high (75-100%) while the SARI positivity rates in Belarus were low. These findings may suggest a selection or testing bias. Furthermore, the observed differences in influenza positivity rates may be explained by some countries reporting SARI all year round and others reporting a selected period in time, and by the age distribution of SARI patients. In general most SARI patients were reported in the young age group (0–15 yrs of age) while the positivity rates were generally higher in the 15+ age group.

Differences in health-seeking behaviour may also have affected the rate of SARI between countries. In a country with many outpatient visit patients, there may be an over-capacity of the secondary and tertiary systems which encourages over-utilisation [21]. Furthermore, the proportion of positive samples may be affected by the sensitivity of the test used, the sample type used and quality of obtaining the specimen, time of sampling after onset of symptoms and the patient groups sampled. Furthermore, the high proportion of negative samples might indicate the presence of other respiratory viruses or bacteria. It would be useful to test negative samples for other pathogens as well if the resources for this are available.

It should be borne mind that data in this study have been collected as part of national surveillance systems. The surveillance is usually performed on a voluntary basis. Therefore limited time may be available and this could affect the quality and completeness of the data collection. Also the healthcare systems of the countries included in this study have different structures and the lack of electronic recording systems in some countries may have limited collection of data.

Despite the limitations of this study a total of nine countries had established SARI surveillance and collected patient data and influenza status, which provides more insight into the role of influenza in hospitalised SARI cases. The findings can be used to compare the severity of influenza by season and country, and address strengths and weaknesses of the current surveillance.

Furthermore, the assessment of possible risk factors for a severe outcome could be improved by performing the analysis by influenza type and subtype. Continued evaluation and review of these recently established SARI surveillance systems will improve our ability to understand these systems and may allow for better comparability of the surveillance data for SARI cases in the WHO European Region.

## Conclusions

SARI surveillance has been successfully implemented in countries in the WHO European Region since 2009. This relatively new system provides a valuable tool for gaining a better understanding of the contribution of influenza infection to the burden of disease. In this study, a total of nine countries located in Central and Eastern Europe provided case-based data on SARI patients. An exploratory analysis was performed on data from Albania, Georgia and Romania resulting in identification of risk factors for ICU admission and death in influenza-positive SARI patients. The heterogeneous results may implicate differences in surveillance and healthcare systems.

Public-health surveillance systems need to be evaluated to ensure they are efficient and effective. SARI surveillance in the European Region is relatively new, implemented during the pandemic season, so the assessment of these systems will help to identify strengths and weaknesses in the current data collection and surveillance activities.

Previously, only very limited information was available on severe influenza in Eastern and Central Europe and this study fills in a gap. The majority of the countries included in this study are low-middle income countries with a low vaccination uptake and antiviral use. Information on risk factors in influenza-positive SARI patients will help in targeting and prioritising vulnerable populations for vaccination and antiviral treatment in these countries. Furthermore, this is a first step towards routine monitoring of SARI in hospitals in Europe and leads to a better understanding of the impact of influenza at the severe spectrum of the disease.

## Additional file

Additional file 1:
**Supplement.**
