# Supplementary material for: Surveillance for severe acute respiratory infections (SARI) in hospitals in the WHO European region - an exploratory analysis of risk factors for a severe outcome in influenza-positive SARI cases
Source: BMC Infect Dis. 2015 Jan 8;15:1. doi: 10.1186/s12879-014-0722-x (PMC4314771; doi:10.1186/s12879-014-0722-x)
Supplement: Additional file 1: — Supplement. [file 12879_2014_722_MOESM1_ESM.docx]

**Supplement**

This supplement contains country tables presenting descriptive data and results of the regression analysis. The countries are ordered alphabetically.

***Table A*** Characteristics of SARI patients for not tested, negative and influenza-positive cases.

***Table B*** Characteristics of influenza-positive SARI patients that are non-severe, admitted to ICU non-fatal, fatal cases and any severe outcome.

***Table C*** Univariate logistic regression analysis - Association risk factor for three severity levels (p-value, OR and 95% CI) for influenza-positive cases.

***Table D*** Multivariate logistic regression analysis -for countries that have data on fatal cases and ICU admission.

**Table 10** Pooled data analyses - Association risk factor for three severity outcomes (10-I) and multivariate risk analysis (10-II) for influenza-positive SARI patients.

**ALBANIA**

**Table 1 (A)**. Characteristics of SARI patients in Albania (n=102). Data were collected in the winter season from week 40-20.

| **Risk Factor** | **SARI patients -**  **influenza**  **negative**  **by season**  **n (%)** | | **SARI patients -**  **influenza**  **positive**  **by season**  **n (%)** | | **Total SARI patients -**  **influenza**  **positive**  **n (%)** |
| --- | --- | --- | --- | --- | --- |
|  | ***Nov 2009-Mar 2010*** | ***Dec 2010- Mar 2011*** | ***Nov 2009-***  ***Mar 2010*** | ***Dec 2010-***  ***Mar 2011*** | ***Nov 2009-***  ***Mar 2011*** |
| **Total SARI patients (n)** | **0** | **33** | **58** | **11** | **69** |
|  |  |  |  |  |  |
| **Age (year)** |  |  |  |  |  |
| < 2 |  | 6/33 (18.2%) | 5/58 (8.6%) | 0/11 (0%) | 5/69 (7.2%) |
| 2 to < 5 |  | 2/33 (6.1%) | 2/58 (3.4%) | 1/11 (9.1%) | 3/69 (4.3%) |
| 5 to < 15 |  | 2/33 (6.1%) | 5/58 (8.6%) | 1/11 (9.1%) | 6/69 (8.7%) |
| 15 to < 25 |  | 6/33 (18.2%) | 9/58 (15.5%) | 1/11 (9.1%) | 10/69 (14.5%) |
| 25 to < 50 |  | 9/33 (27.3%) | 23/58 (39.7%) | 2/11 (18.2%) | 25/69 (36.2%) |
| 50 to < 65 |  | 3/33 (9.1%) | 13/58 (22.4%) | 3/11 (27.3%) | 16/69 (23.2%) |
| ≥65 |  | 5/33 (15.2%) | 1/58 (1.7%) | 3/11 (27.3%) | 4/69 (5.8%) |
| *Median Age (range)* |  | *26 (0-76)* | *33 (0-72)* | *56 (2-87)* | *34 (0-87)* |
| **Gender (% female)** |  | 10/33 (30.3%) | 26/58 (44.8%) | 3/11 (27.3%) | 29/69 (42%) |
| **Underlying medical condition** |  | 18/33 (54.5%) | 25/58 (43.1%) | 6/11 (54.5%) | 31/69 (44.9%) |
| Asthma |  | 0/33 (0%) | 0/58 (0%) | 0/11 (0%) | 0/69 (0%) |
| Diabetes |  | 1/33 (3.0%) | 1/58 (1.7%) | 0/11 (0%) | 1/69 (1.4%) |
| Heart disease |  | 1/33 (3.0%) | 1/58 (1.7%) | 0/11 (0%) | 1/69 (1.4%) |
| Lung disease |  | 18/33 (54.5%) | 23/58 (39.7%) | 5/11 (45.5%) | 28/69 (40.6%) |
| Kidney disease |  | 0/33 (0%) | 0/58 (0%) | 0/11 (0%) | 0/69 (0%) |
| Liver disease |  | 0/33 (0%) | 0/58 (0%) | 0/11 (0%) | 0/69 (0%) |
| Neurological disease |  | 0/33 (0%) | 0/58 (0%) | 0/11 (0%) | 0/69 (0%) |
| Immune compromised |  | 0/33 (0%) | 0/58 (0%) | 1/11 (9.1%) | 1/69 (1.4%) |
| Cancer |  | 0/33 (0%) | 0/58 (0%) | 0/11 (0%) | 0/69 (0%) |
| **Pregnant** |  | 0/6 (0%) | 2/16 (12.5%) | - | 2/16 (12.5%) |
| First trimester |  | - | 0/16 (0%) | - | 0/16 (0%) |
| Second trimester |  | - | 0/16 (0%) | - | 0/16 (0%) |
| Third trimester |  | - | 1/16 (6.25%) | - | 1/16 (6.25%) |
| Pregnant but unknown trimester |  | - | 1/16 (6.25%) | - | 1/16 (6.25%) |
| **Obesity** |  | 0/33 (0%) | 1/58 (1.7%) | 0/11 (0%) | 1/69 (1.4%) |
| BMI 30 – 40 |  | - | - | - | - |
| BMI > 40 |  | - | 1/58 (1.7%) | 0/11 (0%) | 1/69 (1.4%) |
| No BMI measured but judged  clinically |  | - | - | - | - |
| **≥ 2 underlying medical conditions** |  | 2/33 (6.1%) | 2/58 (3.4%) | 0/11 (0%) | 2/69 (2.9%) |
| **Other factors of concern** |  |  |  |  |  |
| Tuberculosis |  | Na | Na | Na | Na |
| **Outcome** |  |  |  |  |  |
| Non-ICU |  | 15/33 (45.5%) | 26/58 (44.8%) | 9/11 (81.8%) | 35/69 (50.7%) |
| ICU |  | 18/33 (54.5%) | 32/58 (55.2%) | 2/11 (18.2%) | 34/69 (49.3%) |
| Deaths |  | 0/33 (0%) | 13/58 (22.4%) | 0/11 (0%) | 13/69 (18.8%) |
| **Cause of death** |  |  |  |  |  |
| Not influenza |  | - | - | - | - |
| Influenza primary cause |  | - | - | - | - |
| Influenza secondary cause |  | - | 13/13 (100%) | - | 13/13 (100%) |
| Not known |  | - | - | - | - |
| **Respiratory support** |  |  |  |  |  |
| Oxygen |  | 1/33 (3.0%) | 13/58 (22.4%) | 0/11 (0%) | 13/69 (18.8%) |
| Ventilation |  | - | - | - | - |
| Both O2 and ventilation |  | - | - | - | - |
| No support required |  | 32/33 (97%) | 45/58 (77.6%) | 11/11 (100%) | 56/69 (81.2%) |
| **Diagnosis pneumonia** |  |  |  |  |  |
| based on clinical symptoms |  | 12/33 (36.4%) | 34/58 (58.6%) | 6/11 (54.5%) | 40/69 (58.0%) |
| **Influenza vaccination received** (within 14 days before symptom onset) |  | 0/33 (0%) | 1/58 (1.7%) | 0/11 (0%) | 1/69 (1.4%) |
| **Antiviral treatment** received <48 hrs after onset |  |  |  |  |  |
| Oseltamivir |  | 7/33 (21.2%) | 8/58 (13.8%) | 2/11 (18.2%) | 10/69 (14.5%) |
| Zanamivir |  | - | - | - | - |
| Oseltamivir and zanamivir |  | - | - | - | - |
| No antiviral treatment provided |  | 26/33 (78.8%) | 50/58 (86.2%) | 9/11 (81.2%) | 59/69 (85.5%) |

Na: not available

**Table 1 (B).** Characteristics of influenza-positive SARI patients that are non-severe, admitted to ICU non-fatal, fatal cases and any severe outcome in Albania.

| **Severity level**  **Risk Factor** | **Total number of**  **non-severe cases**  **(%)** | **Total Cases Requiring Intensive care, non-fatal**  **(%)** | **Fatal cases**  **(%)** | **Any severe outcome (fatal and/or ICU)**  **(%)** |
| --- | --- | --- | --- | --- |
|  |  |  |  |  |
| **Total SARI patients (n)** | **35** | **21** | **13** | **34** |
|  |  |  |  |  |
| **Influenza type/subtype** |  |  |  |  |
| Influenza A not subtyped | - | - | - | - |
| Influenza A(H1N1)pdm | 31/35 (88.6%) | 20/21 (95.2%) | 12/13 (92.3%)( | 32/34 (94.1%) |
| Influenza A(H3N2) | 4/35 (11.4%) | 0/21 (0%) | 1/13 (7.7%) | 1/34 (2.9%) |
| Influenza B | 0/35 (0%) | 1/21 (4.8%) | 0/13 (0%) | 1/34 (2.9%) |
| **Age (year)** |  |  |  |  |
| < 2 | 5/35 (14.3%) | 0/21 (0%) | 0/13 (0%) | 0/34 (0%) |
| 2 to < 5 | 1/35 (2.9%) | 2/21 (9.5%) | 0/13 (0%) | 2/34 (5.9%) |
| 5 to < 15 | 5/35 (14.3%) | 1/21 (4.8%) | 0/13 (0%) | 1/34 (2.9%) |
| 15 to < 25 | 4/35 (11.4%) | 5/21 (23.8%) | 1/13 (7.7%) | 6/34 (17.6%) |
| 25 to < 50 | 11/35 (31.4%) | 9/21 (42.9%) | 5/13 (38.5%) | 14/34 (41.2%) |
| 50 to < 65 | 5/35 (14.3%) | 4/21 (19.0%) | 7/13 (53.8%) | 11/34 (32.3%) |
| ≥65 | 4/35 (11.4%) | 0/21 (0%) | 0/13 (0%) | 0/34 (0%) |
| *Median Age (range)* | *30 (0-87)* | *34 (2-62)* | *50 (18-63)* | *40 (2-63)* |
| **Gender (% female)** | 15/35 (42.9%) | 7/21 (33.3%) | 7/13 (53.8%) | 14/34 (41.2%) |
| **Underlying medical condition** | 10/35 (28.6%) | 10/21 (47.6%) | 11/13 (84.6%) | 21/34 (61.8%) |
| Lung disease | 8/35 (22.9%) | 10/21 (47.6%) | 10/13 (76.9%) | 20/34 (58.8%) |
| Asthma | 0/35 (0%) | 0/21 (0%) | 0/13 (0%) | 0/34 (0%) |
| Diabetes | 0/35 (0%) | 0/21 (0%) | 1/13 (7.7%) | 1/34 (2.9%) |
| Heart disease | 1/35 (2.9%) | 0/21 (0%) | 0/13 (0%) | 0/34 (0%) |
| Kidney disease | 0/35 (0%) | 0/21 (0%) | 0/13 (0%) | 0/34 (0%) |
| Liver disease | 0/35 (0%) | 0/21 (0%) | 0/13 (0%) | 0/34 (0%) |
| Neurological disease | 0/35 (0%) | 0/21 (0%) | 0/13 (0%) | 0/34 (0%) |
| Immune compromised | 1/35 (2.9)% | 0/21 (0%) | 0/13 (0%) | 0/34 (0%) |
| Cancer | 0/35 (0%) | 0/21 (0%) | 0/13 (0%) | 0/34 (0%) |
| **Pregnancy** | 0/8 (0%) | 0/5 (0%) | 2/3 (66.7%) | 2/8 (25%) |
| First trimester | 0/8 (0%) | 0/5 (0%) | 0/3 (0%) | 0/8 (0%) |
| Second trimester | 0/8 (0%) | 0/5 (0%) | 0/3 (0%) | 0/8 (0%) |
| Third trimester | 0/8 (0%) | 0/5 (0%) | 1/3 (33.3%) | 1/8 (12.5%) |
| Pregnant but unknown trimester | 0/8 (0%) | 0/5 (0%) | 1/3 (33.3%) | 1/8 (12.5%) |
| **Obesity** |  |  |  |  |
| BMI 30 – 40 | 0/35 (0%) | 0/21 (0%) | 0/13 (0%) | 0/34 (0%) |
| BMI > 40 | 0/35 (0%) | 0/21 (0%) | 1/13 (7.7%) | 1/34 (2.9%) |
| No BMI measured but judged clinically | 0/35 (0%) | 0/21 (0%) | 0/13 (0%) | 0/34 (0%) |
| **≥ 2 underlying medical conditions** | 0/35 (0%) | 0/21 (0%) | 2/13 (15.4%) | 2/34 (5.9%) |
| **Other factors of concern** |  |  |  |  |
| Tuberculosis | Na | Na | Na | Na |
| **Antiviral treatment** received <48 hrs after onset |  |  |  |  |
| Oseltamivir | 3/35 (8.6%) | 5/21 (23.8%) | 2/13 (15.4%) | 7/34 (20.6%) |
| Zanamivir | - | - | - | - |
| Oseltamivir + zanamivir | - | - | - | - |
| No antiviral treatment provided | 32/35 (91.4%) | 16/21 (76.2%) | 11/13 (84.6%) | 27/34 (79.4%) |
| **Vaccination status** (within 14 days before symptom onset) | 1/35 (2.9%) | 0/21 (0%) | 0/13 (0%) | 0/34 (0%) |

Na: not available

**Table 1 (C).** Univariate logistic regression - Association risk factor for three severity levels (p-value, OR and 95% CI) for influenza-positive cases. Risk factors with p < 0.15 are presented here.

|  | **ICU vs non-ICU**  **and non-fatal**  **(n=56)** | **FATAL vs non-ICU**  **and non-fatal**  **(n=48)** | **ICU/FATAL vs non-ICU and non-fatal (n=69)** |
| --- | --- | --- | --- |
|  |  |  |  |
| Age, 2cat  (0-15, 15+) |  | **OR=1.54**  **(1.22-1.95)**  **p=0.023^a^** | **OR=4.74**  **(1.19-18.88)**  **p=0.042^b^** |
| Underlying condition  (no/yes) |  | OR=13.75  (2.57-73.46)  p=0.002^b^ | OR=4.04  (1.47-11.07)  p=0.011^b^ |
| ≥2 underlying conditions  (no/yes) |  | **OR=2.91**  **(1.30-6.48)**  **p=0.069^a^** |  |
| Lung disease  (no/yes) | OR=3.07  (0.96-9.83)  p=0.078^a^ | **OR=11.25**  **(2.48-51.04)**  **p=0.002^a^** | **OR= 4.82**  **(1.70-13-69)**  **p=0.005^b^** |

**^a^** Fisher exact test – used in case cells have expected count less than 5; ^b^ Continuity Correction – used for 2x2 tables; ^c^ Chi-squared test

**Note:** Results marked in bold were included in the multivariate analysis. Since the variable underlying conditions was strongly correlated with lung disease (R=0.915), we excluded having underlying conditions from the multivariate analysis.

**Table 1 (D).** Multivariate logistic regression.

|  | **ICU vs non-ICU**  **and non-fatal**  **(n=56)** | **FATAL vs non-ICU and non-fatal**  **(n=48)** | **ICU/FATAL vs non-ICU and non-fatal**  **(n=69)** |
| --- | --- | --- | --- |
|  |  |  |  |
| Age, 2cat  (0-15, 15+) | - | p=0.999 | p=0.999 |
| ≥2 underlying conditions  (no/yes) | - | p=0.999 | - |
| Lung disease  (no/yes) | - | **OR=5.33**  **(1.10-25.76)**  **p=0.037** | **OR=5.42**  **(1.29-22.69)**  **p=0.021** |

**ARMENIA**

**Table 2 (A)**. Characteristics of SARI patients in Armenia (n=335). Data were collected all year round.

| **Risk Factor** | **SARI patients -**  **not tested**  **for influenza**  **by season**  **n (%)** | | **SARI patients -**  **influenza**  **negative**  **by season**  **n (%)** | | **SARI patients -**  **influenza**  **positive**  **by season**  **n (%)** | | **Total SARI patients -**  **influenza**  **positive**  **n (%)** |
| --- | --- | --- | --- | --- | --- | --- | --- |
|  | ***Dec 2010 - Jul 2011*** | ***Aug 2011 -***  ***Jul 2012*** | ***Dec 2010 -***  ***Jul 2011*** | ***Aug 2011-***  ***Jul 2012*** | ***Dec 2010 - Jul 2011*** | ***Aug 2011-Jul 2012*** | ***Dec 2010 –***  ***Jul 2012*** |
| **Total SARI patients (n)** | **41** | **106** | **118** | **47** | **22** | **1** | **23** |
|  |  |  |  |  |  |  |  |
| **Age (year)** |  |  |  |  |  |  |  |
| < 2 | 31/41 (75.6%) | 60/106 (56.6%) | 65/118 (55.1%) | 21/47 (44.7%) | 6/22 (27.3%) | 0/1  0%) | 6/23  (26.1%) |
| 2 to < 5 | 7/41 (17.1%) | 26/106 (24.5%) | 18/118 (15.3%) | 7/47 (14.9%) | 2/22  (9.1%) | 0/1  (0%) | 2/23  (8.7%) |
| 5 to < 15 | 3/41  (7.3%) | 12/106 (11.3%) | 18/118 (15.3%) | 11/47 (23.4%) | 0/22  (0%) | 0/1  (0%) | 0/23  (0%) |
| 15 to < 25 | 0/41  (0%) | 4/106 (3.8%) | 4/118 (3.4%) | 3/47  (6.4%) | 5/22 (22.7%) | 0/1  (0%) | 5/23  (21.7%) |
| 25 to < 50 | 0/41  (0%) | 0/106  (0%) | 9/118 (7.6%) | 2/47  (4.3%) | 7/22 (31.8%) | 1/1  (100%) | 8/23  (34.8%) |
| 50 to < 65 | 0/41  (0%) | 2/106 (1.9%) | 3/118 (2.5%) | 2/47  (4.3%) | 2/22  (9.1%) | 0/1  (0%) | 2/23  (8.7%) |
| ≥65 | 0/41  (0%) | 2/106 (1.9%) | 1/118 (0.8%) | 1/47  (2.1%) | 0/22  (0%) | 0/1  (0%) | 0/23  (0%) |
| *Median Age (range)* | *1 (0-9)* | *1 (0-74)* | *1 (0-68)* | *3 (0-88)* | *21 (0-57)* | *45* | *22 (0-57)* |
| **Gender (% female)** | 16/41 (39%) | 48/106 (45.3%) | 44/118 (37.3%) | 23/47 (48.9%) | 12/22 (54.5%) | 1/1  (100%) | 13/23  (56.5%) |
| **Underlying medical condition** | 0/41  (0%) | 7/106 (6.6%) | 7/118* (5.9%) | 4/47*  (8.5%) | 9/22* (59.1%) | 0/1  (0%) | 9/23*  (39.1%) |
| Asthma | - | 3/106 (2.8%) | 0/118  (0%) | 0/47  (0%) | 0/22  (0%) | 0/1  (0%) | 0/23  0%) |
| Diabetes | - | 2/106 (1.9%) | 0/118  (0%) | 0/47  (0%) | 0/22  (0%) | 0/1  (0%) | 0/23  (0%) |
| Heart disease | - | 1/106 (0.9%) | 1/118 (0.8%) | 0/47  (0%) | 0/22  (0%) | 0/1  (0%) | 0/23  (0%) |
| Lung disease | - | 0/106  (0%) | 0/64* | 1/31* | 0/4* | 0/1  (0%) | 0/5* |
| Kidney disease | - | Na | Na | Na | Na | Na | Na |
| Liver disease | - | 0/106  (0%) | 2/93* | 0/43  (0%) | 0/7* | 0/1  (0%) | 0/8* |
| Neurological disease | - | Na | 3/3* | 2/2* | 1/1* | 0/1  (0%) | 1/1* |
| Immune compromised | - | 0/106  (0%) | 0/118  (0%) | 0/47  (0%) | - | 0/1  (0%) | 1/23  (4.3%) |
| Cancer | - | Na | 1/1* | Na | 1/22  (4.5%) | - | Na |
| **Pregnant** |  | 3/3 (100%) | 0/7 (0%) | 1/3 (33%) | 8/10 (80%) | 0/1 (0%) | 8/11 (73%) |
| First trimester | - | 1/3 (33%) | - | - | 1/10 (10%) | - | 1/11 (9.9%) |
| Second trimester | - | 2/3 (67%) | - | 1/3 (33%) | 3/10 (30%) | - | 3/11 7.3%) |
| Third trimester | - | - | - | - | 4/10 (40%) | - | 4/11 (36.4%) |
| **Obesity** | - | - | - | - | - | - | - |
| **≥ 2 underlying medical conditions** | - | 1/106 (0.9%) | 0/118  (0%) | 0/47  (0%) | 1/22  (4.5%) | - | 1/23  (4.3%) |
| **Other factors of concern** |  |  |  |  |  |  |  |
| Tuberculosis | 0/41  (0%) | 0/106  (0%) | 1/118 (0.8%) | 0/47  (0%) | 0/22  (0%) | 0/1  (0%) | 0/23  (0%) |
| **Outcome** |  |  |  |  |  |  |  |
| Non-ICU | 40/41 (97.6%) | 99/106 (93.4%) | 40/118 (33.9%) | 28/47 (59.6%) | 10/22 (45.5%) | 0/1  (0%) | 10/23  (43.5%) |
| ICU | 1/41  (2.4%) | 7/106 (6.6%) | 78/118 (66.1%) | 19/47 (40.4%) | 12/22 (54.5%) | 1/1  (100%) | 13/23  (56.5%) |
| Deaths | 0/41  (0%) | 1/106 (0.9%) | 5/118 (4.2%) | 3/47  (6.4%) | 1/22  (4.5%) | 0/1  (0%) | 1/23  (4.3%) |
| **Cause of death** |  |  |  |  |  |  |  |
| Not influenza | - | 1/1 (100%) | 4/5 (80%) | 1/3 (33.3%) | 1/1 (100%) | - | 1/1 (100%) |
| Influenza primary cause | - | - | - | - | - | - | - |
| Influenza secondary cause | - | - | - | - | - | - | - |
| Not known | - | - | 1/5 (20%) | 2/3 (66.7%) | - | - | - |
| **Respiratory support** |  |  |  |  |  |  |  |
| Oxygen | - | - | 1/104* | - | - | - | 0/10* |
| Ventilation | - | 1/106 (0.9%) | 10/104* | 6/43 (14.0%) | 2/13 (22.2%) | - | 2/10* |
| Both O2 and ventilation | - | - | - | - | - | - | - |
| No support required | 41/41  (0%) | 105/106 (99.1%) | 93/104* | 37/43 (86.0%) | 7/13 (77.8%) | 1/1  (100%) | 8/10* |
| **Diagnosis pneumonia** |  |  |  |  |  |  |  |
| based on clinical symptoms | 41/41 (100%) | 71/106 (67%) | 47/118 (39.8%) | 28/47 (59.6%) | 5/22 (22.7%) |  | 5/23  (21.7%) |
| abnormal chest X-ray |  | 2/106 (1.9%) | 64/118 (54.2%) | 14/47 (29.8%) | 10/22 (45.5%) |  | 10/23 (43.5%) |
| **Influenza vaccination received** (within 14 days before symptom onset) | Na | Na | Na | Na | Na | Na | Na |
| **Antiviral treatment** received <48 hrs after onset |  |  |  |  |  |  |  |
| Oseltamivir | - | - | - | 1/47  (2.1%) | 8/22 (36.4%) | - | 8/23  (34.8%) |
| Zanamivir | - | - | - | - | - | - | - |
| Oseltamivir + zanamivir | - | - | - | - | - | - | - |
| No antiviral treatment provided | 41/41 (100%) | 106/106 (100%) | 118/118 (100%) | 46/47 (97.9%) | 14/22 (63.6%) | 1/1  (100%) | 15/23  (65.2%) |

Na: not available; * > 10% incomplete data

**Table 2 (B).** Characteristics of influenza-positive SARI patients that are non-severe, admitted to ICU non-fatal, fatal cases and any severe outcome in Armenia.

| **Severity level**  **Risk Factor** | **Total number of**  **non-severe cases**  **(%)** | **Total Cases Requiring Intensive care, non-fatal**  **(%)** | **Fatal**  **cases**  **(%)** | **Any severe outcome (fatal and/or ICU)**  **(%)** |
| --- | --- | --- | --- | --- |
|  |  |  |  |  |
| **Total SARI patients (n)** | **10** | **12** | **1** | **13** |
|  |  |  |  |  |
| **Influenza type/subtype** |  |  |  |  |
| Influenza A not subtyped | 5/10 (50%) | 3/12 (25%) | 0/1 (0%) | 3/13 (23.1%) |
| Influenza A(H1N1)pdm | 3/10 (30%) | 6/12 (50%) | 0/1 (0%) | 6/13 (26.1%) |
| Influenza A(H3N2) | 0/10 (0%) | 1/12 (8.3%) | 0/1 (0%) | 1/13 (7.7%) |
| Influenza B | 2/10 (20%) | 2/12 (16.7%) | 1/1 (100%) | 3/13 (23.1%) |
| **Age (year)** |  |  |  |  |
| < 2 | 2/10 (20%) | 3/12 (25.0%) | 1/1 (100%) | 4/13 (30.8%) |
| 2 to < 5 | 1/10 (10%) | 1/12 (8.3%) | 0/1 (0%) | 1/13 (7.7%) |
| 5 to < 15 | 0/10 (0%) | 0/12 (0%) | 0/1 (0%) | 0/13 (0%) |
| 15 to < 25 | 4/10 (40%) | 1/12 (8.3%) | 0/1 (0%) | 1/13 (7.7%) |
| 25 to < 50 | 3/10 (30%) | 5/12 (41.7%) | 0/1 (0%) | 5/13 (38.5%) |
| 50 to < 65 | 0/10 (0%) | 2/12 (16.7%) | 0/1 (0%) | 2/13 (15.4%) |
| ≥65 | 0/10 (0%) | 0/12 (0%) | 0/1 (0%) | - |
| *Median Age (range)* | *21 (1-27)* | *33.5 (1-57)* | *0* | *29 (0-57)* |
| **Gender (% female)** | 9/10 (90%) | 4/12 (33.3%) | 0/1 (0%) | 4/13 (30.8%) |
| **Underlying medical condition** | 7/10 (70%) | 2/12 (16.7%) | 0/1 (0%) | 2/13 (15.4%) |
| Lung disease | 0/3* | 0/12 | - | - |
| Asthma | 0/10 | 0/12 | 0/1 | - |
| Diabetes | 0/10 | 0/12 | 0/1 | - |
| Heart disease | 0/10 | 0/12 | 0/1 | - |
| Kidney disease | Na | Na | Na | Na |
| Liver disease | 0/3* | 0/3* | - | 0/3* |
| Neurological disease | Na | 1/1* | - | 1/1* |
| Immune compromised | 1/10 (10%) | - | 0/1 | 0/1* |
| Cancer | Na | Na | Na | Na |
| **Pregnancy** | 7/7 (100%) | 1/4 (25%) | - | 1/4 (25%) |
| First trimester | 1/7 (14.3%) | 0/4 (0%) | - | 0/4 (0%) |
| Second trimester | 3/7 (42.85%) | 0/4 (0%) | - | 0/4 (0%) |
| Third trimester | 3/7 (42.85%) | 1/4 (25%) | - | 1/4 (25%) |
| Pregnant but unknown  trimester | 0/7 (0%) | 0/4 (0%) | - | 0/4 (0%) |
| **Obesity** | Na | Na | Na | Na |
| **≥ 2 underlying medical conditions** | 1 /10  (10%) | 0/12  (0%) | 0/1  (0%) | 0/13  (0%) |
| **Other factors of concern** |  |  |  |  |
| Tuberculosis | 0/10 (0%) | 0/12 (0%) | 0/1 (0%) | 0/13 (0%) |
| **Antiviral treatment** received <48 hrs after onset |  |  |  |  |
| Oseltamivir | 2/10 (20%) | 5/12 (41.7%) | 1/1 (100%) | 6/13 (46.1%) |
| Zanamivir | - | - | - | - |
| Oseltamivir + zanamivir | - | - | - | - |
| No antiviral treatment provided | 8/10 (80%) | 7/12 (58.3%) | 0/1 (0%) | 7/13 (53.8%) |
| **Vaccination status** (within 14 days before symptom onset) | Na | Na | Na | Na |

Na: not available; * > 10% incomplete data

**BELARUS**

**Table 3 (A)**. Characteristics of SARI patients in Belarus (n=1015). Data are collected all year round.

| **Risk Factor** | **SARI patients -**  **influenza**  **negative**  **by season**  **n (%)** | | **SARI patients -**  **influenza**  **positive**  **by season**  **n (%)** | | **Total SARI patients -**  **influenza**  **positive**  **n (%)** |
| --- | --- | --- | --- | --- | --- |
|  | ***Sep 2010-***  ***Jul 2011*** | ***Aug 2011-***  ***Jul 2012*** | ***Sep 2010-***  ***Jul 2011*** | ***Aug 2011-***  ***Jul 2012*** | ***Sep 2010-***  ***Jul 2012*** |
|  |  |  |  |  |  |
| **Total SARI patients (n)** | **345** | **619** | **35** | **16** | **51** |
|  |  |  |  |  |  |
| **Age (year)** |  |  |  |  |  |
| < 2 | 35/345 (10.1%) | 113/619 (18.3%) | 3/35 (8.6%) | 4/16 (25%) | 7/51 (13.7%) |
| 2 to < 5 | 47/345 (13.6%) | 145/619 (23.4%) | 5/35 (14.3%) | 4/16 (25%) | 9/51 (17.6%) |
| 5 to < 15 | 13/345 (3.8%) | 40/619 (6.5%) | 2/35 (5.7%) | 0/16 (0%) | 2/51 (3.9%) |
| 15 to < 25 | 29/345 (8.4%) | 40/619 (6.5%) | 2/35 (5.7%) | 2/16 (12.5%) | 4/51 (7.8%) |
| 25 to < 50 | 104/345 (30.1%) | 143/619 (23.1%) | 9/35 (25.7%) | 1/16 (6.3%) | 10/51 (19.6%) |
| 50 to < 65 | 94/345 (27.2%) | 97/619 (15.7%) | 11/35 (31.4%) | 4/16 (25%) | 15/51 (29.4%) |
| ≥65 | 23/345 (6.7%) | 41/619 (6.6%) | 3/35 (8.6%) | 1/16 (6.3%) | 4/51 (7.8%) |
| *Median Age (range)* | *37 (0-88)* | *18 (0-92)* | *40 (0-72)* | *10.5 (0-85)* | *34 (0-85)* |
| **Gender (% female)** | 149/345 (43.2%) | 227/608 (45.6%) | 10/35 (28.6%) | 8/15 (53.3%) | 18/50 (36.0%) |
| **Underlying medical condition** | 54/84* | 73/588 (12.4%) | 5/5* | 0/16 (0%) | 5/51 (9.8%) |
| Asthma | 3/3* | 2/588 (0.3%) | - | 0/16 (0%) | 0/16* |
| Diabetes | 6/9* | 11/588 (1.9%) | - | 0/16 (0%) | 0/16* |
| Heart disease | 32/35* | 30/588 (5.1%) | 2/2* | 0/16 (0%) | 2/18* |
| Lung disease | 10/13* | 18/588 (3.1%) | - | 0/16 (0%) | 0/16* |
| Kidney disease | 1/4* | 5/588 (0.9%) | - | 0/16 (0%) | 0/16* |
| Liver disease | 5/7* | 6/588 (1.0%) | 1/1* | 0/16 (0%) | 1/17* |
| Neurological disease | 3/6* | 1/588 (0.2%) | 1/1* | 0/16 (0%) | 1/17* |
| Immune compromised | 0/3* | 11/588 (1.9%) | - | 0/16 (0%) | 0/16* |
| Cancer | 1/4* | 2/588 (0.3%) | - | 0/16 (0%) | 0/16* |
| **Pregnant** | 2/48 (4.2%) | 3/81 (3.7%) | 1/5 (20%) | 0/1 (0%) | 1/6 (16.7%) |
| First trimester | 0/48 (0%) | 1/81 (1.2%) | 1/5 (20%) | 0/1 (0%) | 1/6 (16.7%) |
| Second trimester | 1/48 (2.1%) | 1/81 (1.2%) | 0/5 (0%) | - | - |
| Third trimester | 0/48 (0%) | 0/81 (0%) | 0/5 (0%) | - | - |
| Pregnant but unknown trimester | 1/48 (2.1%) | 1/81 (1.2%) | 0/5 (0%) | - | - |
| **Obesity** | 5/8* | 1/588 (0.5%) | - | 0/16 (0%) | 0 /16* |
| **≥ 2 underlying medical conditions** | 9/84* | 13/588 (2.2%) | 0/35 (0%) | 0/16 (0%) | 0/51 (0%) |
| **Other factors of concern** |  |  |  |  |  |
| Tuberculosis | 1/343 (99.7%) | 0/619 (0%) | 0/35 (0%) | 0/16 (0%) | 0/51 (0%) |
| **Outcome** |  |  |  |  |  |
| Non-ICU | 292/345 (84.6%) | 488/578 (84.4%) | 26/35 (74.3%) | 15/16 (93.8%) | 41/51 (80.4%) |
| ICU | 53/345 (15.4%) | 90/578 (15.6%) | 9/35 (25.7%) | 1/16 (6.3%) | 10/51 (19.6%) |
| Deaths | Na | Na | 0/35 (0%) | Na | Na |
| **Respiratory support** |  |  |  |  |  |
| Oxygen | - | - | - | - | - |
| Ventilation | - | 3/493* | - | - | *-* |
| Both O2 and ventilation | - | - | - | - | - |
| No support required | 3/3* | - | - | 15/15 (100%) | Na* |
| **Diagnosis pneumonia** |  |  |  |  |  |
| based on clinical symptoms | 1/3* | 467/619 (75.5%) | Na | 12/16 (75%) | 1/51* |
| **Influenza vaccination received** (within 14 days before symptom onset) | 9/278* | 8/563  (1.4%) | 1/35  (2.9%) | 0/16  (0%) | 1/5* |
| **Antiviral treatment** received <48 hrs after onset |  | Na* | Na* | Na* | Na |

Na: Not available, * >10% incomplete data; *Note: The total number of SARI cases was 1025, but there were missing data on the age and/or season variables for 10 cases and therefore excluded from the table.*

**Table 3 (B).** Characteristics of influenza-positive SARI patients that are non-severe, admitted to ICU non-fatal in Belarus. No data are available on fatal cases.

| **Severity level**  **Risk Factor** | **Total number of**  **non-severe**  **cases**  **(%)** | **Total Cases Requiring Intensive care**  **(%)** |
| --- | --- | --- |
|  |  |  |
| **Total SARI patients (n)** | **41** | **10** |
|  |  |  |
| **Influenza type/subtype** |  |  |
| Influenza A not subtyped | - | - |
| Influenza A(H1N1)pdm | 18/41 (43.9%) | 7/10 (70%) |
| Influenza A(H3N2) | 14/41 (34.1%) | 1/10 (10%) |
| Influenza B | 9/41 (22.0%) | 2/10 (20%) |
| **Age (year)** |  |  |
| < 2 | 2/26* | 1/9 (11.1%) |
| 2 to < 5 | 5/26* | 0/9 (0%) |
| 5 to < 15 | 1/26* | 1/9 (11.1%) |
| 15 to < 25 | 1/26* | 1/9 (11.1%) |
| 25 to < 50 | 5/26* | 4/9 (44.4%) |
| 50 to < 65 | 9/26* | 2/9 (22.2%) |
| ≥65 | 3/26* | 0/9 (0%) |
| *Median Age (range)* | *26 (0-72)* | *35 (0-85)* |
| **Gender (% female)** | 7/26* | 3/9 (30%) |
| **Underlying medical condition** | 3/26* | 2/9 (22.2%) |
| Lung disease | - | - |
| Asthma | - | - |
| Diabetes | - | - |
| Heart disease | 2/26 (8)* | - |
| Kidney disease | - | - |
| Liver disease | - | 1/9 (11.1%) |
| Neurological disease | - | 1/9 (11.1%) |
| Immune compromised | - | - |
| Cancer | - | - |
| **Pregnancy** | 1/4 (25%) | 0/2 (0%) |
| First trimester | 1/4 (25%) | - |
| Second trimester | - | - |
| Third trimester | - | - |
| Pregnant but unknown trimester | - | - |
| **Obesity** | Na | Na |
| **≥ 2 underlying medical conditions** | 0/26* | 0/9 (0%) |
| **Other factors of concern** |  |  |
| Tuberculosis | 0/26* | 0/9 (0%) |
| **Antiviral treatment** received <48 hrs after onset | 4/26* | 2/9 (22.2%) |
| **Vaccination status** (within 14 days before symptom onset) | 1/26* | 0/9 (0%) |

Na: Not available, * >10% incomplete data

**GEORGIA**

**Table 4 (A)**. Characteristics of SARI patients in Georgia (n=2137). Data were generally collected during the winter season.

| **Risk Factor** | **SARI patients -**  **influenza**  **negative**  **by season**  **n (%)** | | | | **SARI patients -**  **influenza**  **positive**  **by season**  **n (%)** | | | | **Total SARI patients -**  **influenza**  **positive**  **n (%)** |
| --- | --- | --- | --- | --- | --- | --- | --- | --- | --- |
|  | ***Jan 2009-Jul 2009*** | ***Aug 2009-Apr 2010*** | ***Nov 2010-May 2011*** | ***Sep 2011-Apr 2012*** | ***Jan 2009- Jul 2009*** | ***Aug 2009-Apr 2010*** | ***Nov 2010-May 2011*** | ***Sep 2011-Apr 2012*** | ***Jan***  ***2009-***  ***Apr***  ***2012*** |
| **Total SARI patients (n)** | **153** | **1009** | **93** | **11** | **46** | **533** | **282** | **10** | **871** |
|  |  |  |  |  |  |  |  |  |  |
| **Age (year)** |  |  |  |  |  |  |  |  |  |
| < 2 | 70/150 (46.7%) | 354/995 (35.6%) | 20/93 (21.5%) | 0/11  (0%) | 2/46 (4.3%) | 74/531 (13.9%) | 45/282 (16%) | 1/10 (10%) | 122/870 (14.0%) |
| 2 to < 5 | 33/150 (22%) | 151/995 (15.2%) | 15/93 (16.1%) | 0/11  (0%) | 7/46 (15.2%) | 24/531 (4.5%) | 28/282 (9.9%) | 1/10 (10%) | 60/870 (6.9%) |
| 5 to < 15 | 24/150 (16%) | 132/995 (13.3%) | 5/93 (5.4%) | 1/11 (9.1%) | 19/46 (41.3%) | 107/531 (20.2%) | 24/282 (8.5%) | 0/10 (0%) | 150/870 (17.2%) |
| 15 to < 25 | 3/150 (2.0%) | 87/995 (8.7%) | 6/93 (6.5%) | 1/11 (9.1%) | 5/46 (10.9%) | 122/531 (23%) | 53/282 (18.8%) | 2/10 (20%) | 183/870 (21.0%) |
| 25 to < 50 | 13/150 (8.7%) | 143/995 (14.4%) | 21/93 (22.6%) | 6/11 (54.5%) | 8/46 (17.4%) | 154/531 (29%) | 72/282 (25.5%) | 2/10 (20%) | 236/870 (27.1%) |
| 50 to < 65 | 4/150 (2.7%) | 73/995 (7.3%) | 11/93 (11.8%) | 2/11 (18.2%) | 5/46 (10.9%) | 38/531 (7.2%) | 30/282 (10.6%) | 0/10  (0%) | 73/870 (8.4%) |
| ≥65 | 3/150 (2%) | 55/995 (5.5%) | 15/93 (16.1%) | 1/11 (9.1%) | 0/46 (0%) | 12/531 (2.3%) | 30/282 (10.6%) | 4/10 (40%) | 46/870 (5.3%) |
| *Median Age (range)* | *2*  *(0-88)* | *4*  *(0-88)* | *25*  *(0-80)* | *32*  *(13-77)* | *10*  *(1-58)* | 19  (0-90) | *23*  *(0-95)* | *34*  *(0-78)* | *20*  *(0-95)* |
| **Gender (% female)** | 67/153 (43.8%) | 433/1008 (43%) | 56/92 (60.9%) | 8/11 (80%) | 19/46 (41.3%) | 255/528 (48.3%) | 150/282 (53.2%) | 3/10 (30%) | 428/867 (49.4%) |
| **Underlying medical condition** | 27/153 (17.6%) | 95/1009 (9.4%) | 13/93 (14%) | 1/11 (9.1%) | 6/46 (13%) | 63/533 (11.8%) | 145/282 (51.4%) | 7/10 (70%) | 221/871 (25.4%) |
| Asthma | 0/150 (0%) | 9/1009 (0.9%) | 0/93 (0%) | 1/11 (9.1%) | 0/46 (0%) | 7/533 (1.3%) | 2/282 (0.7%) | 0/10 (0%) | 9/871 (1.0%) |
| Diabetes | 3/153 (2%) | 5/1009 (0.5%) | 3/93 (3.2%) | 0/11 (0%) | 0/46 (0%) | 14/533 (2.6%) | 11/282 (3.9%) | 2/10 (20%) | 25/871 (2.9%) |
| Heart disease | 3/153 (2%) | 30/1009 (3.0%) | 4/93 (4.3%) | 0/11 (0%) | 2/46 (4.3%) | 5/533 (0.9%) | 66/282 (23.4%) | 5/10 (50%) | 78/871 (9.0%) |
| Lung disease | 14/153 (9.2%) | 27/1009 (2.7%) | 6/93 (6.5%) | 0/11 (0%) | 3/46 (6.5%) | 40/533 (7.5%) | 105/282 (37.2%) | 2/10 (20%) | 150/871 (17.2%) |
| Kidney disease | 0/153 (0%) | 10/1009 (1.0%) | 2/93 (2.2%) | 0/11 (0%) | 0/46 (0%) | 4/533 (0.8%) | 7/282 (2.5%) | 2/10 (20%) | 13/871 (1.5%) |
| Liver disease | 0/153 (0%) | 5/1009 (0.5%) | 1/93 (1.1%) | 0/11 (0%) | 0/46 (0%) | 3/533 (0.6%) | 3/282 (1.1%) | 0/10 (0%) | 6/871 (0.7%) |
| Neurological disease | 3/153 (2%) | 3/1009 (0.3%) | 2/93 (2.2%) | 1/11 (9.1%) | 0/46 (0%) | 1/533 (0.2%) | 4/282 (1.4%) | 1/10 (10%) | 6/871 (0.7%) |
| Immune compromised | 1/153 (0.7%) | 0/1009 (0%) | 0/93 (0%) | 0/11 (0%) | 0/46 (0%) | 0/533 (0%) | 0/282 (0%) | 0/10 (0%) | 0/871  (0%) |
| Cancer | 1/153 (0.7%) | 0/1009 (0%) | 0/93 (0%) | 0/11 (0%) | 0/46 (0%) | 1/533 (0.2%) | 2/282 (0.7%) | 0/10 (0%) | 3/871 (0.3%) |
| **Pregnant** | 0/10 (0%) | 4/125 (3.3%) | 1/20 (5%) | 0/6 (0%) | 0/4 (0%) | 11/133 (8.3%) | 20/66 (30.3%) | 1/1 (100%) | 32/205 (15.6%) |
| First trimester | - | - | - | - | - | 2/133  (1.5%) | 4/66  (6.1%) | 1/1  (100%) | 7/205 (3.4%) |
| Second trimester | - | 1/125 (0.8%) | - | - | - | 4/133  (3.0%) | 10/66  (15.2%) | - | 14/205 (6.8%) |
| Third trimester | - | - | - | - | - | - | - | - | - |
| Pregnant but unknown trimester /missing | - | 3/125  (2.4%) | 1/20 (5%) | - | - | 5/133  (3.8%) | 6/66 (9.1%) | - | 11/20  (5.4%) |
| **Obesity** | Na | 1/1* | Na | Na | Na | 8/9* | 8/236* | 1/6* | 17/251* |
| BMI 30 – 40 | - | - | - | - | - | 0/9* | 3/236* | 0/6* | 3/251* |
| BMI > 40 | - | - | - | - | - | - | - | - | - |
| No BMI measured  but judged  clinically | - | 1/1* | - | - | - | 8/9* | 5/236* | 1/6* | 14/251* |
| **≥ 2 underlying medical conditions** | 5/153 (3.3%) | 17/1009 (1.7%) | 4/93 (4.3%) | 0/11 (0%) | 0/46 (0%) | 26/533 (4.9%) | 66/282 (23.4%) | 4/10 (40%) | 96/871 (11.0%) |
| **Other factors of concern** |  |  |  |  |  |  |  |  |  |
| Tuberculosis | 0/153 (0%) | 1/1009 (0.1%) | 0/93 (0%) | 0/11 (0%) | 0/46 (0%) | 1/533 (0.2%) | 1/282 (0.4%) | 0/10 (0%) | 2/871 (0.2%) |
| **Outcome** |  |  |  |  |  |  |  |  |  |
| Non-ICU | 143/143 (100%) | 602/652* | 67/67* | 11/11 (100%) | 45/45 (100%) | 308/ 345* | 58/277 (20.9%) | 3/10 (30%) | 414/677 (61.2%)* |
| ICU | 0/143  (0%) | 50/  652* | 0/67* | 0/11 (0%) | 0/45 (0%) | 37/345* | 219/277 (79.1%) | 7/10 (70%) | 263/677 (38.8%)* |
| Deaths | 0/153 (0%) | 0/1009 (0%) | 0/93 (0%) | 0/11 (0%) | 0/46 (0%) | 31/533 (5.8%) | 53/282  (18.8%) | 7/10 (70%) | 92/872 (10.6%) |
| **Cause of death** | - | - | - | - | - | - | - | - | - |
| **Respiratory support** |  |  |  |  |  |  |  |  |  |
| Oxygen | 0/143  (0%) | 2/666* | - | 0/10  (0%) | 0/45  (0%) | 1/  353* | 4/269 (1.5%) | 0/10 (0%) | 5/  677* |
| Ventilation | 0/143  (0%) | 71/666* | - | 0/10  (0%) | 0/45  (0%) | 57/  353* | 147/269 (54.6%) | 7/10 (70%) | 211/  677* |
| Both O2 and ventilation | 0/143  (0%) | 0/666* | - | 0/10  (0%) | 0/45  (0%) | 0/  353* | 0/269  (0%) | 0/10  (0%) | 0/  677* |
| No support required | 143/143 (100%) | 0/666* | - | 10/10  (100%) | 45/45 (100%) | 295/  353* | 118/269 (43.9%) | 3/10 (30%) | 461  /677* |
| **Diagnosis pneumonia** |  |  |  |  |  |  |  |  |  |
| based on clinical symptoms | 1/153 (0.7%) | 100/1008 (9.9%) | 1/93 (1.1%) | 0/11 (0%) | 1/46 (2.2%) | 65/532 (12.2%) | 110/282 (39%) | 7/10 (70%) | 183/870 (21.0%) |
| **Influenza vaccination received** (within 14 days before symptom onset) | 0/153 (0%) | 1/1009 (0.1%) | 0/93 (0%) | 0/11 (0%) | 0/46 (0%) | 0/533 (0%) | 0/282 (0%) | 0/10 (0%) | 0/871  (0%) |
| **Antiviral treatment** received <48 hrs after onset | 8/153 (4.6%) | 44**/  1009 (4.4%) | 2**/93 (2.2%) | 0/11 (0%) | 0/46 (0%) | 47**/533 (8.8%) | 59**/282 (20.9%) | 3/9 (33%) | 109**/871 (12.5%) |
| Oseltamivir | 8/153 (5.2%) | 40/993 (4%) | Na | - | - | 29/532 (5/5%) | 44/281 (15.7%) | - | 73/868 (8.4%) |
| Zanamivir | - | - | Na | - | - | - | 2/281 (3.2%) | - | 2/868 (0.2%) |
| Oseltamivir + zanamivir | - | - | Na | - | - | - | 9/281 (3.2%) | 3/9 (33.3%) | 12/868 (1.4%) |
| No antiviral treatment provided | 145/153 (94.8%) | 948/993 (95.5%) | 91/93 (97.8%) | 11/11 (100%) | 46/46 (100%) | 486/533 (91.2%) | 223/282 (79.1%) | 6/9 (66.7%) | 761/870  (87.7%) |

Na: Not available, * >10% incomplete data, ** includes other (not specified) type of treatment.

**Table 4 (B).** Characteristics of influenza-positive SARI patients that are non-severe, admitted to ICU non-fatal, fatal cases and any severe outcome in Georgia.

| **Severity level**  **Risk Factor** | **Total number of**  **non-severe cases**  **(%)** | **Total cases in ICU, non-fatal**  **(%)** | **Fatal**  **cases**  **(%)** | **Any severe outcome (fatal and/or ICU) (%)** |
| --- | --- | --- | --- | --- |
|  |  |  |  |  |
| **Total SARI patients (n)** | **399** | **192** | **92** | **284** |
|  |  |  |  |  |
| **Influenza type/subtype** |  |  |  |  |
| Influenza A not subtyped | 3/399 (0.8%) | 0/192 (0%) | 1/92 (1.1%) | 1/284 (0.3%) |
| Influenza A(H1N1)pdm | 347/399 (87.0%) | 84/192 (43.8%) | 65/92 (70.7%) | 149/284 (52.5%) |
| Influenza A(H3N2) | 12/399 (3.0%) | 0/192 (0%) | 5/92 (5.4%) | 5/284 (1.8%) |
| Influenza B | 37/399 (9.3%) | 108/192 (56.2%) | 21/92 (22.8%) | 129/284 (45.4%) |
| **Age (year)** |  |  |  |  |
| < 2 | 75/399 (18.8%) | 41/192 (21.4%) | 0/92 (0%) | 41/284 (14.4%) |
| 2 to < 5 | 23/399 (5.8%) | 24/192 (12.5%) | 3/92 (3.2%) | 27/284 (9.5%) |
| 5 to < 15 | 89/399 (22.3%) | 15/192 (7.8%) | 2/92 (2.2%) | 17/284 (6.0%) |
| 15 to < 25 | 81/399 (20.3%) | 45/192 (23.4%) | 7/92 (7.6%) | 52/284 (18.3%) |
| 25 to < 50 | 93/399 (23.3%) | 43/192 (22.4%) | 40/92 (43.5%) | 83/284 (29.2%) |
| 50 to < 65 | 30/399 (7.5%) | 16/192 (8.3%) | 13/92 (14.1%) | 29/284 (10.2%) |
| ≥65 | 8/399 (2.0%) | 8/192 (4.2%) | 27/92 (29.3%) | 35/284 (12.3%) |
| *Median Age (range)* | *16 (0-90)* | *18 (0-87)* | *47 (2-95)* | *25 (0-95)* |
| **Gender (% female)** | 209/397 (52.6%) | 99/192 (51.6%) | 47/92 (51.1%) | 146/284 (51.4%) |
| **Underlying medical condition** | 44/399 (11.0%) | 78/192 (40.6%) | 88/91 (96.7%) | 166/283 (58.7%) |
| Lung disease | 20/399 (5.0%) | 57/192 (29.7%) | 68/91 (74.7%) | 125/283 (44.1%) |
| Asthma | 5/399 (1.25%) | 0/192 (0%) | 4/91 (4.4%) | 4/283 (1.4%) |
| Diabetes | 8/399 (2.0%) | 3/192 (1.6%) | 12/91 (13.2%) | 15/283 (5.3%) |
| Heart disease | 4/399 (2.9% | 29/192 (15.1%) | 44/91 (48.3%) | 27/283 (9.5%) |
| Kidney disease | 1/399 (0.2%) | 1/192 (0.5%) | 9/91 (9.9%) | 10/283 (3.5%) |
| Liver disease | 2/399 (0.5%) | 0/192 (0%) | 4/91 (4.4%) | 4/283 (1.4%) |
| Neurological disease | 2/399 (0.5%) | 0/192 (0%) | 4/91 (4.4%) | 4/283 (1.4%) |
| Immune compromised | 0/399 (0%) | 0/192 (0%) | 0/91 (0%) | 0/283 (0%) |
| Cancer | 1/399 (0.2%) | 0/192 (0%) | 2/91 (2.2%) | 2/283 (0.7%) |
| **Pregnancy** | 6/128 (4.7%) | 15/70 (21.4%) | 10/27 (37.0%) | 25/93 (26.9%) |
| **Obesity** | 3/22* | 3/166* | 10/62* | 13/228* |
| **≥ 2 underlying medical conditions** | 10/399 (2.5%) | 24/192 (12.5%) | 60/92 (65.2%) | 84/284 (29.6%) |
| **Other factors of concern** |  |  |  |  |
| Tuberculosis | 0/399 (0%) | 0/192 (0%) | 2/91 (2.2%) | 2/228* |
| **Antiviral treatment** received <48 hrs after onset |  |  |  |  |
| Type of treatment unknown | 24/399 (6.0%) | 19/192 (9.9%) | 58/91 (63.7%) | 77/283 (27.2%) |
| **Vaccination status** (within 14 days before symptom onset) | 0/399 (0%) | 0/192 (0%) | 0/91 (0%) | 0/283 (0%) |

Na: Not available, * >10% incomplete data

**Table 4 (C).** Univariate logistic regression - Association risk factor for ICU admission and outcome (p-value, OR and 95% CI) for influenza-positive cases. Risk factors with p < 0.15, marked in bold, are included in the multivariate logistic regression analysis.

|  | **ICU vs non-ICU**  **and non-fatal**  **(n=591)** | **FATAL vs non-ICU**  **and non-fatal**  **(n=491)** | **ICU/FATAL vs non-ICU and non-fatal**  **(n=677)** |
| --- | --- | --- | --- |
| Age, 2cat  (0-15, 15+) |  | **OR=13.64**  **(7.18-25.91)**  **p=0.000^b^** | **OR=2.17**  **(1.58-2.97)**  **p=0.000^b^** |
| Age, 4cat  (<5, 5-14, 15-49, 50+) | p=0.014^c^ | p=0.000^c^ | p=0.000^c^ |
| Age, 7cat  (<2, 2-5, 5-14, 14-29, 30-49, 50-64, 65+) | p=0.000^c^ | p=0.000^c^ | p=0.000^c^ |
| Underlying condition  (no/yes) | OR=5.520  (3.61-8.45)  p=0.000^b^ | OR=236.6  (71.81-779.9)  p=0.000^b^ | OR=11.43  (7.71-16.95)  p=0.000^b^ |
| >2 underlying conditions  (no/yes) | OR=5.56  (2.60-11.87)  p=0.000^b^ | OR=72.93  (34.09-156.0)  p=0.000^b^ | OR=16.27  (8.26-32.08)  p=0.000^b^ |
| Asthma  (no/yes) |  | **p=0.066^a^**  **OR=3.62**  **(0.95-13.76)** |  |
| Diabetes  (no/yes) |  | **OR=7.42**  **(2.94-18.74)**  **p=0.000^b^** | **OR=2.40**  **(0.98-5.86)**  **p=0.000^b^** |
| Obesity  (no/yes) | **OR=0.12**  **(0.02-0.62)**  **p=0.022^a^** |  |  |
| Lung conditions  (no/yes) | **OR= 8.00**  **(4.64-13.81)**  **p=0.000^b^** | **p=0.000^b^**  **OR=56.02**  **(29.17-107.5)** | **p=0.000^b^**  **OR=15.03**  **(9.047-24.99)** |
| Cancer  (no/yes) |  | **OR=8.944**  **(0.80-99.72)**  **P=0.090^a^** |  |
| Heart disease  (no/yes) | **OR=16.86**  **(5.82-48.82)**  **p=0.000^b^** | **OR=92.44**  **(31.79-268.7)**  **P=0.000^b^** |  |
| Liver disease  (no/yes) |  | **OR=9.13**  **(1.65=50.62)**  **p=0.012^b^** |  |
| Kidney disease  (no/yes) |  | **OR=43.68**  **(5.46-349.5)**  **p=0.000^a^** | **OR=14.85**  **(1.89-116.6)**  **p=0.001^a^** |
| Neurological disease  (no/yes) |  | **OR=9.13**  **(1.65-50.62)**  **p=0.012^a^** |  |
| Pregnant  (no/yes) | **OR=5.55**  **(2.12-14.64)**  **p=0.000^b^** | **OR = 7.99**  **(2.82-22.59)**  **p=0.000^a^** | **OR=5.91**  **(2.37-14.70)**  **p=0.000^b^** |
| TB (no TB vs pos test hosp) |  | **OR=0.18**  **(0.15-0.22)**  **p=0.034^a^** |  |
| A(H1N1)pdm09  (no/yes) |  | **OR=0.36**  **(0.21-0.62)**  **p=0.000^b^** |  |

**^a^** Fisher exact test – used in case cells have expected count less than 5; ^b^ Continuity Correction – used for 2x2 tables; ^c^ Chi-squared test

**Note:** Results marked in bold were included in the multivariate analysis. Since the variable age category (0-14, 15+) was strongly correlated with the other age group categories and the variable having underlying conditions was strongly correlated with specific underlying diseases, we excluded these variable from the multivariate analysis.

**Table 4 (D).** Multivariate logistic regression.

|  | **ICU vs non-ICU and non-fatal**  **(n=591)** | **FATAL vs non-ICU and non-fatal**  **(n=490)** | **ICU/FATAL vs non-ICU and non-fatal (n=677)** |
| --- | --- | --- | --- |
| Age cat (0-14, 15+) |  | **OR=10.73**  **(3.80-30.28)**  **p=0.000** | **OR=9.75**  **(4.61-20.61)**  **p=0.000** |
| Asthma (no/yes) |  | p=0.858 |  |
| Diabetes (no/yes) |  | p=0.364 | p=0.152 |
| Lung disease  (no/yes) | **OR=5.94**  **(3.35-10.58)**  **p=0.000** | **OR=43.65**  **(16.45-115.7)**  **p=0.000** | **OR=14.90**  **(8.14-27.27)**  **p=0.000** |
| Cancer  (no/yes) |  | p=0.527 |  |
| Heart disease  (no/yes) | **OR=10.47**  **(3.45-31.83)**  **p=0.000** | **OR=40.62**  **(8.34-198.0)**  **p=0.000** |  |
| Liver disease  (no/yes) |  | p=0.851 |  |
| Kidney disease  (no/yes) |  | p=0.322 | **OR=8.78**  **(1.50-51.29)**  **p=0.016** |
| Neurological disease  (no/yes) |  | p=0.591 |  |
| Pregnant  (no/yes) | **OR=6.02**  **(2.22-16.33)**  **p=0.000** | **OR=18.39**  **(3.92-86.38)**  **p=0.000** | **OR=3.27**  **(1.13-9.49)**  **p=0.029** |
| TB (no TB vs pos test hosp) |  | p=0.999 |  |
| A(H1N1)pdm09  (no/yes) |  | p=0.709 |  |

**Kazakhstan**

**Table 5 (A).** Characteristics of SARI patients in Kazakhstan (n=814). Data were collected from Sep 2011-Dec 2012 all year round. This was considered 1 season.

| **Risk Factor** | **SARI patients -**  **not tested for influenza**  **n (%)** | **SARI patients -influenza**  **negative**  **n (%)** | **SARI patients -**  **influenza**  **positive**  **n (%)** |
| --- | --- | --- | --- |
|  | ***Sep 2011-***  ***Dec 2012*** | ***Sep 2011-***  ***Dec 2012*** | ***Sep 2011-***  ***Dec 2012*** |
| **Total SARI patients (n)** | **23** | **605** | **186** |
|  |  |  |  |
| **Age (year)** |  |  |  |
| < 2 | 7/23 (30.4%) | 205/605 (33.9%) | 33/186 (17.7%) |
| 2 to < 5 | 3/23 (13.0%) | 172/605 (28.4%) | 42/186 (22.6%) |
| 5 to < 15 | 1/23 (4.3%) | 37/605 (6.1%) | 20/186 (10.8%) |
| 15 to < 25 | 7/23 (30.4%) | 96/605 (15.9%) | 44/186 (23.7%) |
| 25 to < 50 | 2/23 (8.7%) | 71/605 (11.7%) | 40/186 (21.5%) |
| 50 to < 65 | 1/23 (4.3%) | 21/605 (3.5%) | 5/186 (2.7%) |
| ≥65 | 2/23 (8.7%) | 3/605 (0.5%) | 2/186 (1.1%) |
| *Median Age (range)* | *15 (1-74)* | *2 (1-84)* | *13 (1-85)* |
| **Gender (% female)** | 8/23 (34.8%) | 233/605 (38.5%) | 90/186 (48.4%) |
| **Underlying medical condition** | 3/23 (13.0%) | 85/605 (14.0%) | 47/186 (25.3%) |
| Asthma | 0/23 (0%) | 2/605 (0.3%) | 2/186 (1.1%) |
| Diabetes | 0/23 (0%) | 0/605 (0%) | 0/186 (0%) |
| Heart disease | 1/23 (4.3%) | 6/605 (1.0%) | 3/186 (1.6%) |
| Lung disease | 0/23 (0%) | 9/605 (1.5%) | 1/186 (0.5%) |
| Kidney disease | 0/23 (0%) | 6/605 (1.0%) | 5/186 (2.7%) |
| Liver disease | 0/23 (0%) | 3/605 (0.5%) | 0/186 (0%) |
| Neurological disease | 2/23 (8.7%) | 5/605 (0.8%) | 2/186 (1.1%) |
| Immune compromised | 0/23 (0%) | 0/605 (0%) | 0/186 (0%) |
| Cancer | 0/23 (0%) | 0/605 (0%) | 1/186 (0.5%) |
| **Pregnant** |  |  |  |
| Pregnant but unknown trimester | 1/4 (25%) | 32/88 (36.4%) | 27/58 (46.6%) |
| **Obesity** |  |  |  |
| BMI 30 – 40 | 0/23 (0%) | 5/605 (0.8%) | 2/186 (1.1%) |
| **≥ 2 underlying medical conditions** | 1/23 (4.3%) | 6/605 (0.9%) | 4/186 (2.2%) |
| **Other factors of concern** |  |  |  |
| Tuberculosis | Na | Na | Na |
| **Outcome** |  |  |  |
| Non-ICU | 22/23 (95.7%) | 580/605 (95.9%) | 174/186 (93.5%) |
| ICU | 1/23 (4.3%) | 25/605 (4.1%) | 12/186 (6.5%) |
| Deaths | 0/17* | 0/274* | 0/38* |
| **Respiratory support** |  |  |  |
| Oxygen | 1/23 (4.3%) | 8/605 (1.3%) | 2/186 (1.1%) |
| Ventilation | - | - | - |
| Both O2 and ventilation | - | - | - |
| No support required | 22/23 (95.7%) | 597/605 (98.7%) | 188/186 (98.9%) |
| **Diagnosis pneumonia** |  |  |  |
| based on clinical symptoms | Na | Na | Na |
| **Influenza vaccination received** (within 14 days before symptom onset) | 2/23 (8.7%) | 7/605 (1.2%) | 2/186 (1.1%) |
| **Antiviral treatment** received <48 hrs after onset |  |  |  |
| Oseltamivir | - | 1/605 (0.2%) | - |
| Zanamivir | - | - | - |
| Other type of treatment | - | 6/605 (1.0%) | 1/186 (0.5%) |
| No antiviral treatment provided | 23/23 (100%) | 598/605 (98.8%) | 185/186 (99.5%) |

Na: Not available, * >10% incomplete data

**Table 5 (B).** Characteristics of influenza-positive SARI patients with non-severe outcome, admission to ICU non-fatal. No information was available on fatal cases.

| **Severity level**  **Risk Factor** | **Total number of**  **non-severe cases**  **(%)** | **Total cases in ICU,**  **non-fatal**  **(%)** |
| --- | --- | --- |
|  |  |  |
| **Total SARI patients** | **174** | **12** |
|  |  |  |
| **Influenza type/subtype** |  |  |
| Influenza A not subtyped | 20 | 2 |
| Influenza A(H1N1)pdm | 50 | 4 |
| Influenza A(H3N2) | 96 | 4 |
| Influenza B | 8 | 2 |
| **Age (year)** |  |  |
| < 2 | 27/174 (15.5%) | 6/12 (50%) |
| 2 to < 5 | 38/174 (21.8%) | 4/12 (33.3%) |
| 5 to < 15 | 19/174 (10.9%) | 1/12 (8.3%) |
| 15 to < 25 | 44/174 (25.3%) | 0/12 (0%) |
| 25 to < 50 | 39/174 (22.4%) | 1/12 (8.3%) |
| 50 to < 65 | 5/174 (2.9%) | 0/12 (0%) |
| ≥65 | 2/174 (1.1%) | 0/12 (0%) |
| *Median Age (range)* | *16 (1-85)* | *2 (1-30)* |
| **Gender (% female)** | 86/174 (49.4%) | 4/12 (33.3%) |
| **Underlying medical condition** | 46/174 (26.4%) | 1/12 (8.3%) |
| Lung disease | 1/174 (0.6%) | 0/12 (0%) |
| Asthma | 2/174 (1.1%) | 0/12 (0%) |
| Diabetes | 0/174 (0%) | 0/12 (0%) |
| Heart disease | 3/174 (1.7%) | 0/12 (0%) |
| Kidney disease | 5/174 (2.9%) | 0/12 (0%) |
| Liver disease | 0/174 (0%) | 0/12 (0%) |
| Neurological disease | 2/174 (1.1%) | 0/12 (0%) |
| Immune compromised | 0/174 (0%) | 0/12 (0%) |
| Cancer | 1/174 (0.6%) | 0/12 (0%) |
| **Pregnancy** | 27/57 (47.4%) | 0/1 (0%) |
| First trimester | - | - |
| Second trimester | - | - |
| Third trimester | - | - |
| Pregnant but unknown trimester | 27/57 (47.4%) | - |
| **Obesity** | 2/174 (1.1%) | 0/12 (0%) |
| BMI 30 – 40 | 2/174 (1.1%) | - |
| BMI > 40 | - | - |
| No BMI measured but judged clinically | - | - |
| **≥ 2 underlying medical conditions** | 4/174 (2.3%) | 0/12 (0%) |
| **Other factors of concern** |  |  |
| Tuberculosis | Na | Na |
| **Antiviral treatment** received <48 hrs after onset | 1/174  (0.6%) | 0/12  (0%) |
| Oseltamivir | - | - |
| Zanamivir | - | - |
| Other treatment | 1/174 (0.6%) | - |
| No treatment | 173/174 (99.4%) | 12/12 (100%) |
| **Vaccination status** (within 14 days before symptom onset) | 2/174  (1.1%) | 0/12  (0%) |

Na: Not available, * >10% incomplete data

**Kyrgyzstan**

**Table 6 (A)**. Characteristics of SARI patients in Kyrgyzstan (n=366). Data were collected from Nov 2010-Jun 2011.

| **Risk Factor** | **SARI patients -**  **not tested for influenza**  **n (%)** | **SARI patients -influenza**  **negative**  **n (%)** | **SARI patients -**  **influenza**  **positive**  **n (%)** |
| --- | --- | --- | --- |
|  | ***Nov 2010-***  ***Jun 2011*** | ***Nov 2010-***  ***Jun 2011*** | ***Nov 2010- Jun 2011*** |
| **Total SARI patients (n)** | **323** | **24** | **19** |
|  |  |  |  |
| **Age (year)** |  |  |  |
| < 2 | 89/322 (27.6%) | 0/24 (0%) | 6/19 (31.6%) |
| 2 to < 5 | 101/322 (31.1%) | 0/24 (0%) | 4/19 (21.1%) |
| 5 to < 15 | 67/322 (20.6%) | 0/24 (0%) | 3/19 (15.8%) |
| 15 to < 25 | 35/322 (10.8%) | 3/24 (12.5%) | 2/19 (10.5%) |
| 25 to < 50 | 17/322 (5.2%) | 3/24 (12.5%) | 2/19 (10.5%) |
| 50 to < 65 | 9/322 (2.8%) | 15/24 (62.5%) | 0/19 (0%) |
| ≥65 | 4/322 (1.2%) | 3/24 (12.5%) | 2/19 (10.5%) |
| *Median Age (range)* | *3 (1-74)* | *54 (18-79)* | *2 (1-74)* |
| **Gender (% female)** | 134/323 (41.5%) | 12/14* | 12/19 (63.2%) |
| **Underlying medical condition** | 7/323 (2.2%) | 11/24 (45.8%) | 2/19 (10.5%) |
| Asthma | 1/323 (0.3%) | 2/24 (8.3%) | 0/19 (0%) |
| Diabetes | 4/319 (1.3%) | 1/24 (4.2%) | 0/19 (0%) |
| Heart disease | 2/319 (0.6%) | 2/24 (12.5%) | 0/19 (0%) |
| Lung disease | 0/319 (0%) | 5/24 (20.8%) | 2/19 (10.5%) |
| Kidney disease | Na | Na | Na |
| Liver disease | Na | Na | Na |
| Neurological disease | Na | Na | Na |
| Immune compromised | 0/323 (0%) | 0/24 (0%) | 0/19 (0%) |
| Cancer | 0/319 (0%) | 1/24 (4.2%) | 0/19 (0%) |
| **Pregnant** |  |  |  |
| Pregnant but unknown trimester | 1/28 (3.6%) | - | - |
| **Obesity** |  |  |  |
| BMI 30 – 40 | 2/323 (0.6%) | Na | 0 |
| **≥ 2 underlying medical conditions** | 1/323 (0.3%) | 1/24 (4.2%) | 0/19 |
| **Other factors of concern** |  |  |  |
| Tuberculosis | 0/1* | 0/24 (0%) | 0/3* |
| **Outcome** |  |  |  |
| Non-ICU | 316/323 (97.8%) | 24/24 (100%) | 19/19 (100%) |
| ICU | 7/323 (2.2%) | 0/24 (0%) | 0/19 (0%) |
| Deaths | 0/2* | 0/24 (0%) | 0/3* |
| **Respiratory support** |  |  |  |
| Oxygen | 1/233* | - | - |
| Ventilation | 1/233* | - | - |
| Both O2 and ventilation | - | - | - |
| No support required | 226/233* | 24/24 | 0/4* |
| **Diagnosis pneumonia** | Na | Na | Na |
| based on clinical symptoms |  |  |  |
| **Influenza vaccination received** (within 14 days before symptom onset) | 0/321 (0%) | 0/24 (0%) | 0/19 (0%) |
| **Antiviral treatment** received <48 hrs after onset | 0/321 (0%) | 0/24 (0%) | 0/19 (0%) |

Na: not available; * > 10% incomplete data; *Note: The total number of SARI cases was 369, but there were missing data on the age and/or season variables for 3 cases and therefore excluded from the table.*

**ROMANIA**

**Table 7 (A).** Characteristics of SARI patients in Romania (n=1003).

| **Risk Factor** | **SARI patients -**  **not tested**  **for influenza**  **by season**  **n (%)** | | **SARI patients -**  **influenza**  **negative**  **by season**  **n (%)** | | | **SARI patients -**  **influenza**  **positive**  **by season**  **n (%)** | | | **Total SARI patients**  **flu+**  **n (%)** |
| --- | --- | --- | --- | --- | --- | --- | --- | --- | --- |
|  | ***Aug 2010-May 2011*** | ***Nov 2011-May 2012*** | ***Oct 2009-May 2010*** | ***Aug 2010-May 2011*** | ***Nov 2011-May 2012*** | ***Oct 2009-May 2010*** | ***Aug 2010-May 2011*** | ***Nov 2011-May 2012*** | ***Oct***  ***2009-***  ***May***  ***2012*** |
| **Total SARI patients (n)** | **25** | **64** | **145** | **257** | **212** | **66** | **165** | **69** | **300** |
|  |  |  |  |  |  |  |  |  |  |
| **Age (year)** |  |  |  |  |  |  |  |  |  |
| < 2 | 3/25 (12%) | 36/64 (56.3%) | 31/145 (21.4%) | 67/257 (26.1%) | 93/212 (43.9% | 5/66 (7.6%) | 15/165 (9.1%) | 13/69 (18.8%) | 33/300 (11.0%) |
| 2 to < 5 | 2/25 (8%) | 19/64 (29.7%) | 19/145 (13.1%) | 40/257 (15.6%) | 40/212 (18.9%) | 3/66 (4.5%) | 16/165 (9.7%) | 10/69 (14.5%) | 29/300  (9.7%) |
| 5 to < 15 | 4/25 (16%) | 6/64 (9.4%) | 12/145 (8.3%) | 27/257 (10.5%) | 9/212 (4.2%) | 5/66 (7.6%) | 38/165 (23%) | 8/69 (11.6%) | 51/300 (17.0%) |
| 15 to < 25 | 3/25 (12%) | 1/64 (1.6%) | 9/145 (6.2%) | 13/257 (5.1%) | 10/212 (4.7%) | 5/66 (7.6%) | 22/165 (13.3%) | 3/69 (4.3%) | 30/300 (10.0%) |
| 25 to < 50 | 8/25 (32%) | 0/64 (0%) | 41/145 (6.2%) | 58/257 (22.6%) | 23/212 (10.8) | 26/66 (39.4%) | 47/165 (28.5%) | 12/69 (17.4%) | 85/300 (28.3%) |
| 50 to < 65 | 1/25 (4%) | 1/64 (1.6%) | 24/145 (16.6%) | 31.257 (12.1%) | 23/212 (10.8%) | 15/66 (22.7%) | 19/165 (11.5%) | 10/69 (14.5%) | 44/300 (14.7%) |
| ≥65 | 4/25 (16%) | 1/64 (1.6%) | 9/145 (6.2%) | 21/257 (8.2%) | 14/212 (6.6%) | 7/66 (10.6%) | 8/165 (4.8%) | 13/69 (18.8%) | 28/300  (9.3%) |
| *Median Age (range)* | *25*  *(0-77)* | *1*  *(0-66)* | *28*  *(0-79)* | *13*  *(0-86)* | *2*  *(0-96)* | *36*  *(0-79)* | *21*  *(0-82)* | *27*  *(0-89)* | *26.5*  *(0-88)* |
| **Gender (% female)** | 9/25 (36%) | 33/64 (51.6%) | 55/145 (37.9%) | 122/257 (47.5%) | 85/212 (40.1%) | 36/66 (54.5%) | 76/165 (46.1%) | 35/69 (50.7%) | 147/300 (49.0%) |
| **Underlying medical condition** | 9/25 (36%) | 9/64 (14.1%) | 75/145 (51.7%) | 113/257 (44%) | 65/212 (30.7%) | 43/66 (65.2%) | 91/165 (55.2%) | 38/69 (55.1%) | 172/300 (57.3%) |
| Asthma | 0/25 (0%) | 5/64 (7.8%) | 13/145 (9%) | 14/257 (5.4%) | 9/212 (4.2%) | 4/66 (6.1%) | 9/165 (5.5%) | 5/69 (7.2%) | 18/300  (6.0%) |
| Diabetes | 0/25 (0%) | 0/64 (0%) | 13/145 (9%) | 17/257 (6.6%) | 5/212 (2.4%) | 4/66 (6.1%) | 10/165 (6.1%) | 4/69 (5.8%) | 18/300  (6.0%) |
| Heart disease | 4/25 (16%) | 3/64 (4.7%) | 40/145 (27.6%) | 58/257 (22.6%) | 34/212 (16%) | 14/66 (21.2%) | 27/165 (16.4%) | 23/69 (33.3%) | 64/300 (21.3%) |
| Lung disease | 3/25 (12%) | 2/64 (3.1%) | 28/145 (19.3%) | 19/257 (7.4%) | 9/212 (4.2%) | 9/66 (13.6%) | 10/165 (6.1%) | 5/69 (7.2%) | 24/300  (8.0%) |
| Kidney disease | 0/25 (0%) | 0/64 (0%) | 0/145 (0%) | 5/257 (1.9%) | 6/212 (2.8%) | 3/66 (4.5%) | 3/165 (1.8%) | 1/69 (1.4%) | 7/300  (2.3%) |
| Liver disease | 0/25 (0%) | 0/64 (0%) | 3/145 (2.1%) | 9/257 (3.5%) | 5/212 (2.4%) | 6/66 (9.1%) | 8/165 (4.8%) | 1/69 (1.4%) | 15/300  (5.0%) |
| Neurological disease | 0/25 (0%) | 0/64 (0%) | 5/145 (3.4%) | 0/257 (0%) | 8/212 (3.8%) | 0/66  (0%) | 0/165 (0%) | 1/69 (1.4%) | 1/300  (0.3%) |
| Immune compromised | 0/25 (0%) | 1/64 (1.6%) | 8/145 (5.5%) | 20/257 (7.8%) | 7/212 (3.3%) | 4/66 (6.1%) | 19/165 (11.5%) | 6/69 (8.7%) | 29/300  (9.7%) |
| Cancer | 1/25 (4%) | 0/64 (0%) | 0/145 (0%) | 3/257 (1.2%) | 1/212 (0.5%) | 0/66  (0%) | 2/165 (1.2%) | 4/69 (5.8%) | 6/300  (2.0%) |
| **Pregnant** |  |  |  |  |  |  |  |  |  |
| Pregnant but unknown trimester | 2/3  (66.7%) | 0/1  (0%) | 2/17 (11.8%) | 6/38 (15.8%) | 4/15 (26.7%) | 3/15 (20%) | 10/32 (31.2%) | 5/11 (45.5%) | 18/58  (31.0%) |
| **Obesity** | 3/25 (12%) | 0/64 (0%) | 21/145 (14.5%) | 26/257 (10.1%) | 12/212 (5.7%) | 17/66 (25.8%) | 25/165 (15.2%) | 9/69  (13.0%) | 51/300 (16.9%) |
| BMI 30 – 40 | 3/25 (12%) | 0/64 | 21/145 (14.5%) | 26/257  (10.1%) | 5/212  (2.4%) | 17/66 (25.8%) | 25/165  15.2%) | 7/69  (10.1%) | 49/300  (16.3%) |
| BMI > 40 | - | - | - | - | - | - | - | 1/69 (1.45%) | 1/300  (0.33%) |
| No BMI measured, judged clinically | - | - | - | - | 7/212 (3.3%) | - | - | 1/69  (1.45%) | 1/300  (0.33%) |
| **≥ 2 underlying medical conditions** | 3/25 (12%) | 2/64 (3.1%) | 43/145 (29.7%) | 45/257 (17.5%) | 24/212 (11.3%) | 13/66 (19.7%) | 26/165 (15.8%) | 18/69 (26.1%) | 57/300 (19.0%) |
| **Other factors of concern** |  |  |  |  |  |  |  |  |  |
| Tuberculosis | 0/25 (0%) | 0/64 (0%) | 0/145 (0%) | 1/257 (0.4%) | 2/212 (0.9%) | 0/66  (0%) | 1/165 (0.6%) | 0/69  (0%) | 1/300  (0.3%) |
| **Outcome** |  |  |  |  |  |  |  |  |  |
| Non-ICU | 20/25 (80%) | 62/64 (96.9%) | 90/142 (63.4%) | 156/257 (60.7%) | 173/212 (18.4%) | 35/66 (53%) | 108/165 (65.5%) | 61/69 (88.4%) | 204/300 (68.0%) |
| ICU | 5/25 (20%) | 2/64 (3.1%) | 52/142 (36.6) | 101/257 (39.3%) | 39/212 (18.4%) | 31/66 (47.0%) | 57/165 (34.5%) | 8/69 (11.6%) | 96/300 (32.0%) |
| Deaths | 1/25 (4%) | 1/64 (1.6%) | 3/145 (2.1%) | 8/257 (3.1%) | 4/212 (1.9%) | 11/66 (16.7%) | 21/165 (12.7%) | 1/69 (1.4%) | 33/300 (11.0%) |
| **Cause of death** |  |  |  |  |  |  |  |  |  |
| Not influenza | 1/1 (100%) | 1/1 (100%) | 1/1* | 1/1* | 4/4 (100%) | 1/11 (9.1%) | - | - | 1/27* |
| Influenza primary cause | - | - | - | - | - | 7/11 (63.6%) | 15/15* | 1/1 (100%) | 23/27* |
| Influenza secondary cause | - | - | - | - | - | 3/11 (27.3%) | - | - | 3/27* |
| **Respiratory support** |  |  |  |  |  |  |  |  |  |
| Oxygen | 9/25 (36%) | 15/64 (23.4%) | 10/145 (6.9%) | 175/257 (68.1%) | 61/212 (28.8%) | 14/66 (21.2%) | 102/165 (61.8%) | 20/69 (29%) | 136/300 (45.3%) |
| Ventilation | 2/25 (8%) | - | - | 16/257 (6.2%) | 16/212 (7.5%) | - | 12/165 (7.3%) | 6/69 (8.7%) | 18/300  (6.0%) |
| Both O2 and ventilation | 2/25 (8%) | - | - | 16/257 (6.2%) | - | - | 12/165 (7.3%) | - | 12/300  (4.0%) |
| No support required | 12/25 (48%) | 49/64 (76.6%) | 135/145 (93.1%) | 50/257 (19.5%) | 135/212 (63.7%) | 52/66 (78.8%) | 39/165 (23.6%) | 43/69 (62.3%) | 134/300 (44.7%) |
| **Diagnosis pneumonia** |  |  |  |  |  |  |  |  |  |
| based on clinical symptoms | 25/25 (100%) | 44/64 (68.8%) | 131/145 (90%) | 240/257 (93.4%) | 165/212 (77.8%) | 55/66 (83.3%) | 139/165 (84.2%) | 49/69 (71%) | 243/300 (81.0%) |
| **Influenza vaccination received** (< 14 days before symptom onset) |  |  |  |  |  |  |  |  |  |
| Seasonal | 1/25 (4%) | 0/64 (0%) | 6/142 (4.2%) | 6/253 (2.4%) | 3/212 (1.4%) | 1/65 (1.5%) | 3/165 (1.8%) | 1/69 (1.4%) | 5/299  (1.7%) |
| Pandemic | 1/25 (4%) | 0/64 (0%) | 3/144 (2.1%) | 2/253 (0.8%) | 0/211 (0%) | 0/66  (0%) | 0/165 (0%) | 0/69  (0%) | 0/300  (0%) |
| **Antiviral treatment** received <48 hrs after onset | 7**/25 (28%) | 1/64 (1.6%) | 0/145 (0%) | 80**/257 (31.1%) | 17**/212 (8%) | 1**/66 (1.5%) | 84**/165 (50.9%) | 13**/69 (18.8%) | 98**/214* |
| Oseltamivir | Na | - | - | Na | 8/209  (3.8%) | Na | Na | 6/68 (8.8%) | 6/214* |
| Zanamivir | Na | 1/64 (1.6%) | - | Na | 4/209 (1.9%) | Na | Na | 5/68 (7.4%) | 5/214* |
| Oseltamivir + zanamivir | Na | - | - | Na | 3/209 (1.4%) | Na | Na | 1/68 (1.5%) | 1/214* |
| No antiviral treatment provided | 18/25 (72%) | 63/64 (98.4%) | 145/145 (100%) | 177/257 (68.9%) | 195/212 (92%) | 65/66 (98.5%) | 81/165 (49.1%) | 56/69 (81.2%) | 202/214* |

Na: not available; * > 10% incomplete data; ** includes other (not specified) type of treatment

**Table 7 (B).** Characteristics of influenza-positive SARI patients with non-severe outcome, admission to ICU non-fatal, fatal cases and any severe outcome in Romania.

| **Severity level**  **Risk Factor** | **Total number of**  **non-severe cases**  **(%)** | **Total Cases Requiring Intensive care, non-fatal**  **(%)** | **Fatal**  **cases**  **(%)** | **Any severe outcome (fatal and/or ICU)**  **(%)** |
| --- | --- | --- | --- | --- |
|  |  |  |  |  |
| **Total SARI patients (n)** | **201** | **66** | **33** | **99** |
|  |  |  |  |  |
| **Influenza type/subtype** |  |  |  |  |
| Influenza A not subtyped | - | - | - | - |
| Influenza A(H1N1)pdm | 84/201 (41.8%) | 36/66 (54.5%) | 29/33 (87.9%) | 65/99 (65.65%) |
| Influenza A(H3N2) | 60/201 (29.85%) | 8/66 (12.1%) | 1/33 (3.0%) | 9/99 (9.1%) |
| Influenza B | 57/201 (28.35%) | 22/66 (33.3%) | 3/33 (9.1%) | 25/99 (25.25%) |
| **Age (year)** |  |  |  |  |
| < 2 | 25/201 (12.4%) | 7/66 (10.6%) | 1/33 (3.0%) | 8/99 (8.1%) |
| 2 to < 5 | 21/201 (10.4%) | 8/66 (12.1%) | 0/33 (0%) | 8/99 (8.1%) |
| 5 to < 15 | 36/201 (17.9%) | 13/66 (19.7%) | 2/33 (6.1%) | 15/99 (15.1%) |
| 15 to < 25 | 22/201 (10.9%) | 5/66 (7.6%) | 3/33 (9.1%) | 8/99 (8.1%) |
| 25 to < 50 | 51/201 (25.4%) | 17/66 (25.8%) | 17/33 (51.5%) | 34/99 (34.3%) |
| 50 to < 65 | 26/201 (12.9%) | 10/66 (15.2%) | 8/33 (24.2%) | 18/99 (18.2%) |
| ≥65 | 20/201 (10.0%) | 6/66 (9.1%) | 2/33 (6.1%) | 8/99 (8.1%) |
| *Median Age (range)* | *23 (0-88)* | *23.5 (0-87)* | *40 (1-77)* | *33 (0-87)* |
| **Gender (% female)** | 98/201 (48.8%) | 30/66 (45.5%) | 19/33 (57.6%) | 49/99 (49.5% ) |
| **Underlying medical condition** | 107/201 (53.2%) | 36/66 (54.5%) | 29/33 (87.9%) | 65/99 (65.7%) |
| Lung disease | 13/201 (6.5%) | 7/66 (10.6%) | 4/33 (12.1%) | 11/99 (11.1%) |
| Asthma | 14/201 (7.0%) | 3/66 (4.5%) | 1/33 (3.0%) | 4/99 (4.0%) |
| Diabetes | 10/201 (5.0%) | 6/66 (9.1%) | 2/33 (6.1%) | 8/99 (8.1%) |
| Heart disease | 43/201 (21.4%) | 11/66 (16.7%) | 10/33 (30.3%) | 21/99 (21.2%) |
| Kidney disease | 4/201 (2.0%) | 1/66 (1.5%) | 2/33 (6.1%) | 3/99 (3.0%) |
| Liver disease | 6/201 (3.0%) | 4/66 (6.1%) | 5/33 (15.2%) | 9/99 (9.1%) |
| Neurological disease | 1/201 (0.5%) | 0/66 (0%) | 0/33 (0%) | 0/99 (0%) |
| Immune compromised | 16/201 (8.0%) | 6/66 (9.1%) | 7/33 (21.2%) | 13/99 (13.1%) |
| Cancer | 5/201 (2.5%) | 1/66 (1.5%) | 0/33 (0%) | 1/99 (1.0%) |
| **Pregnancy** |  |  |  |  |
| Pregnant but unknown trimester | 11/37 (29.7%) | 3/11 (27.3%) | 4/10 (40.0%) | 7/21 (33.3%) |
| **Obesity** |  |  |  |  |
| BMI 30 – 40 | 26/201 (12.9%) | 13/66 (19.7%) | 10/33 (30.3%) | 23/99 (23.2%) |
| BMI > 40 | 1/201 (0.5%) | - | - | - |
| No BMI measured but judged  clinically | 1/201 (0.5%) | - | - | - |
| **≥ 2 underlying medical conditions** | 33/201 (16.4%) | 12/66 (18.2%) | 12/33 (36.4%) | 24/99 (24.2%) |
| **Other factors of concern** |  |  |  |  |
| Tuberculosis | 1/201 (0.5%) | 0/66 (0%) | 0/33 (0%) | - |
| **Antiviral treatment** received <48 hrs after onset | 57/201 (28.4%) | 25/66 (37.9%) | 16/33 (48.5%) | 41/99 (41.1%) |
| Oseltamivir | 4/201 (2%) | 2/66 (3%) | - | 2/99 (2%) |
| Zanamivir | 4/201 (2%) | 1/66 (1.5%) | - | 1/99 (1%) |
| Oseltamivir + zanamivir | - | 1/66 (1.5%) | - | 1/99 (1%) |
| Other not specified treatment/not available | 49/201 (24.3%) | 21/66 (31.8%) | 16/33 | 37/99 (37%) |
| **Vaccination status** (within 14 days before symptom onset) | 2/201 (1.0%) | 2/66 (3.0%) | 1/33 (3%) | 3/99 (3%) |

Na: not available; * > 10% incomplete data

**Table 7 (C).** Univariate logistic regression - Association risk factor (p-value, chi square test) for ICU admission and outcome (p-value, OR and 95% CI) for influenza-positive cases. Risk factors with p < 0.15 are included in the multivariate logistic regression analysis.

|  | **ICU vs non-ICU**  **and non-fatal**  **(n=267)** | **FATAL vs non-ICU**  **and non-fatal**  **(n=234)** | **ICU/FATAL vs non-ICU and non-fatal**  **(n=300)** |
| --- | --- | --- | --- |
| Age, 2cat  (0-15, 15+) |  | **OR=6.81**  **(2.04-23.33)**  **p=0.001^b^** | **OR=1.51**  **(0.91-2.52)**  **p=0.142^b^** |
| Age, 4cat  (<5, 5-14, 15-49, 50+) |  | p=0.005^c^ |  |
| Age, 7cat (<2, 2-5, 5-14, 14-29, 30-49, 50-64, 65+) |  | p=0.007^c^ |  |
| Underlying condition  (no/yes) |  | OR=6.37  (2.16-18.78)  p=0.000^b^ | OR=1.67  (1.02-2.77)  p=0.055^b^ |
| >2 underlying conditions  (no/yes) |  | OR=2.91  (1.304-6.484)  p=0.014^b^ | OR=1.63  (0.90-2.95)  p=0.142 |
| Obesity  (no/yes) |  | **OR=2.69**  **(1.16-6.24)**  **p=0.035^b^** | **OR=1.87**  **(1.01-3.46)**  **p=0.064^b^** |
| Immune  (no/yes) |  | **OR=3.11**  **(1.17-8.28)**  **p=0.027^a^** |  |
| Liver  (no/yes) |  | **OR=5.80**  **(1.66-20.28)**  **p=0.010^a^** | **OR=3.25**  **(1.12-9.41)**  **p=0.044^a^** |
| A(H1N1)pdm09  (no/yes) | **OR=1.67**  **(0.995-2.93)**  **p=0.096** | **OR=10.98**  **(3.42-29.80)**  **p=0.000^b^** | **OR=2.66**  **(1.61-4.39)**  **p=0.000^b^** |

Fisher exact test – used in case cells have expected count less than 5; ^b^ Continuity Correction – used for 2x2 tables; ^c^ Chi-squared test

**Table 7 (D).** Multivariate logistic regression.

|  | **ICU vs non-ICU and non-fatal**  **(n=267)** | **FATAL vs non-ICU and non-fatal**  **(n=234)** | **ICU/FATAL vs non-ICU and non-fatal**  **(n=300)** |
| --- | --- | --- | --- |
| Age cat (0-14, 15+) |  | p=0.128 | p=0.872 |
| Obese  (no/yes) |  | p=0.250 | p=0.232 |
| Immune  (no/yes) |  | **OR=3.87**  **(1.19-12.60)**  **p=0.025** |  |
| Liver disease  (no/yes) |  | p=0.156 | p=0.122 |
| A(H1N1)pdm09  (no/yes) |  | **OR=7.48**  **(2.39-23.36)**  **p=0.001** | **OR=2.40**  **(1.39-4.12)**  **p=0.002** |

**RUSSIAN FEDERATION**

**Table 8 (A).** Characteristics of SARI patients in the Russian Federation (n=2779). Data were collected for the 2010-2011 and 2011-2012 seasons – all year round.

| **Risk Factor** | **SARI patients -**  **influenza**  **negative**  **by season**  **n (%)** | | **SARI patients -**  **influenza**  **positive**  **by season**  **n (%)** | | **Total SARI patients -**  **influenza**  **positive**  **n (%)** |
| --- | --- | --- | --- | --- | --- |
|  | ***Sep 2010-***  ***May 2011*** | ***Aug 2011-Dec 2012*** | ***Sep 2010-May 2011*** | ***Aug 2011-Dec 2012*** | ***Sep 2010-***  ***Dec 2012*** |
| **SARI patients** | **1019** | **1333** | **274** | **153** | **427** |
|  |  |  |  |  |  |
| **Age (year)** |  |  |  |  |  |
| < 2 | 358/1019 (35.1%) | 486/1333 (36.5%) | 27/274 (9.9%) | 10/153 (6.5%) | 37/427 (8.7%) |
| 2 to < 5 | 228/1019 (22.4%) | 238/1333 (17.9%) | 29/274 (10.6%) | 19/153 (12.4%) | 48/427 (11.2%) |
| 5 to < 15 | 145/1019 (14.2%) | 111/1333 (8.3%) | 47/274 (17.2%) | 20/153 (13.1%) | 67/427 (15.7%) |
| 15 to < 25 | 97/1019 (9.5%) | 215/1333 (16.1%) | 65/274 (23.7%) | 37/153 (24.2%) | 102/427 (23.9%) |
| 25 to < 50 | 130/1019 (12.8%) | 223/1333 (16.7%) | 75/274 (27.4%) | 46/153 (30.1%) | 121/427 (28.3%) |
| 50 to < 65 | 38/1019 (3.7%) | 29/1333 (2.2%) | 21/274 (7.7%) | 12/153 (7.8%) | 33/427 (7.7%) |
| ≥65 | 23/1019 (2.3%) | 31/1333 (2.3%) | 10/274 (3.6%) | 9/153 (5.9%) | 19/427 (4.4%) |
| *Median Age (range)* | *3 (0-88)* | *3 (0-89)* | *21.5 (0-88)* | *23 (0-87)* | *22 (0-88)* |
| **Gender (% female)** | 445/1019 (43.7%) | 720/1333 (54%) | 154/274 56.2%) | 99/153 (64.7%) | 253/427 (59.3%) |
| **Underlying medical condition** | 222/1019 (21.8%) | 353/1333 (26.5%) | 113/274 41.2%) | 74/153 (48.4%) | 187/427 (43.8%) |
| Asthma | 9/1019 (0.9%) | 15/1333 (1.1%) | 9/274 (3.3%) | 4/153 (2.6%) | 13/427 (3.0%) |
| Diabetes | 9/1019 (0.9%) | 11/1333 (0.8%) | 2/274 (0.7%) | 2/153 (1.3%) | 4/427 (0.9%) |
| Heart disease | 25/1019 (2.5%) | 32/1333 (2.4%) | 17/274 (6.2%) | 10/153 (6.5%) | 27/427(6.3%) |
| Lung disease | 25/1019 (2.5%) | 23/1333 (1.7%) | 12/274 (4.4%) | 4/153 (2.6%) | 16/427 (3.7%) |
| Kidney disease | 5/5* | 4/4* | Na | Na | Na |
| Liver disease | 9/1019 (0.9%) | 8/1333 (0.6%) | 2/274 (0.7%) | 3/153 (2%) | 5/427 (1.2%) |
| Neurological disease | 8/1019 (0.8%) | 23/1333 (1.7%) | 0/274 (0%) | 0/153 (0%) | 0/427 (0%) |
| Immune compromised | 58/1019 (5.7%) | 32/1333 (2.4%) | 13/274 (4.7%) | 1/153 (0.7%) | 14/427 (3.3%) |
| Cancer | Na | Na | 1/1* | 0/153 (0%) | 1/1* |
| **Pregnancy** |  |  |  |  |  |
| Pregnant but unknown trimester | 91/148 (61.5%) | 217/317 (68.5%) | 65/99 (65.7%) | 53/66 (80.3%) | 118/165 (71.5%) |
| **Obesity** |  |  |  |  |  |
| BMI 30 – 40 | 2/2* | 5/5* | - | - | - |
| **≥ 2 underlying medical conditions** | 19/1019 (1.9%) | 14/1333 (1.1%) | 8/274 (2.9%) | 3/153 (2%) | 11/427 (2.6%) |
| **Outcome** | Na | Na | Na | Na | Na |
| **Respiratory support** | Na | Na | Na | Na | Na |
| **Diagnosis pneumonia** |  |  |  |  |  |
| based on clinical symptoms | 470/989 (47.5%) | 606/1308 (46.3%) | 92/264 (34.8%) | 48/150 (32%) | 140/414 (33.8%) |
| **Other factors of concern** |  |  |  |  |  |
| Tuberculosis | Na | Na | Na | Na | Na |
| **Vaccination status** (within 14 days before symptom onset) | 23/  868* | 25/  1178* | 11/247 (4.5%) | 5/144  (3.5%) | 16/391  (4.1%) |
| **Antiviral treatment** received <48 hrs after onset | Na | Na | Na | Na | Na |

Na: Not available, * >10% incomplete data

**UKRAINE**

**Table 9 (A)**. Characteristics of SARI patients in Ukraine (n=4650). Influenza data are available for season 2009-2010 (Sep 2009-Jul 2010), 2010-2011 (Aug 2010-Jul 2011), and 2011-2012 (Aug 2011-Dec 2012). Data were collected all year round.

| **Risk Factor** | **SARI patients -**  **influenza**  **negative**  **by season**  **n (%)** | | | **SARI patients -**  **influenza**  **positive**  **by season**  **n (%)** | | | **Total SARI patients -**  **influenza**  **positive**  **n (%)** |
| --- | --- | --- | --- | --- | --- | --- | --- |
|  | ***Sep 2009-Jul 2010*** | ***Aug 2010-Jul 2011*** | ***Aug 2011-Dec 2012*** | ***Sep 2009-Jul 2010*** | ***Aug 2010-Jul 2011*** | ***Aug 2011-Dec 2012*** | ***Sep 2009-Dec 2012*** |
| **Total SARI patients (n)** | **1418** | **1556** | **245** | **731** | **490** | **210** | **1431** |
|  |  |  |  |  |  |  |  |
| **Age (year)** |  |  |  |  |  |  |  |
| < 2 | 307/1418 (21.7%) | 391/1556 (25.1%) | 68/225 (30.2%) | 51/731 (7%) | 42/490 (8.6%) | 14/188* (7.4%) | 107/1409 (7.6%) |
| 2 to < 5 | 220/1418 (15.5%) | 296/1556 (19%) | 37/225 (16.4%) | 53/731 (7.3%) | 56/490 (11.4%) | 44/188* (23.4%) | 153/1409 (10.9%) |
| 5 to < 15 | 179/1418 (12.6%) | 239/1556 (15.4%) | 39/225 (17.3%) | 144/731 (19.7% | 110/490 (22.4%) | 49/188* (26.1%) | 303/1409 (21.5%) |
| 15 to < 25 | 256/1418 (18.1%) | 304/1556 (19.5%) | 27/225 (12%) | 235/731 (32.1%) | 157/490 (32%) | 28/188* (14.9%) | 420/1409 (29.8%) |
| 25 to < 50 | 337/1418 (23.8%) | 228/1556 (14.7%) | 36/225 (16%) | 195/731 (26.7%) | 101/490 (20.6%) | 45/188*  (23.9%) | 341/1409 (24.2%) |
| 50 to < 65 | 88/1418 (6.2%) | 42/1556 (2.7%) | 14/225 (6.2%) | 48/731 (6.6%) | 13/490 (2.7%) | 8/188* (4.3%) | 69/1409 (4.9%) |
| ≥65 | 31/1418 (2.2%) | 56/1556 (3.6%) | 4/225  (1.8%) | 5/731 (0.7%) | 11/490 (2.2%) | 0/188*  (0%) | 16/1409 (1.1%) |
| *Median Age (range)* | *15 (0-82)* | *6 (0-100)* | *5 (0-100)* | *19 (0-79)* | *17 (0-100)* | *10 (0-61)* | *18 (0-100)* |
| **Gender (% female)** | 631/1418 (44.5%) | 664/1556 (42.7%) | 108/239 (45.2%) | 310/731 (42.2%) | 236/490 (48.2%) | 107/189 (56.6%) | 653/1431 (45.6%) |
| **Underlying medical condition** | Na | Na | Na | Na | Na | Na | Na |
| Asthma | 2/2* | 2/2* | - | - | 1/1* | - | 1/1* |
| Diabetes | 6/6* | 5/5* | - | - | 2/2* | - | 2/2* |
| Heart disease | 17/17* | 12/12* | - | 3/3* | 3/3* | - | 6/6* |
| Lung disease | 2/2* | 6/6* | - | 1/1* | 5/5* | - | 6/6* |
| Kidney disease | Na | 3/3* | - | - | 1/1* | - | 1/1* |
| Liver disease | 1/1* | 4/4* | - | - | 1/1* | - | 1/1* |
| Neurological disease | Na | Na | - | - | - | - | - |
| Immune compromised | Na | Na | - | - | - | - | - |
| Cancer | Na | Na | - | - | - | - | - |
| **Pregnant** | 0/262  (0%) | 10/201  (5%) | 7/32  (21.9%) | 2/174 (1.1%) | 17/127 (13.4%) | 34/52 (65.4%) | 53/353 (15.0%) |
| First trimester | - | - | - | 0/174  (0%) | 0/127  (0%) | 8/52  (15.4%) | 8/353  (2.3%) |
| Second trimester | - | - | 1/32  (3.1%) | 0/174  (0%) | 0/127  (0%) | 5/52  (9.6%) | 5/353  (1.4%) |
| Third trimester | - | - | 6/32  (18.8%) | 0/174  (0%) | 0/127  (0%) | 17/52  (32.7%) | 17/353  (4.8%) |
| Pregnant but unknown trimester | - | 10/201  (5%) | - | 2/174  (1.1%) | 17/127  (13.4%) | 4/52  (7.7%) | 23/353 (6.5%) |
| **Obesity** | Na | 30/30* | 4/4* | - | 20/20* | - | 20/20* |
| BMI 30 – 40 | - | 30 | 3 | - | 20 | - | 20 |
| BMI > 40 | - | - | - | - | - | - | - |
| No BMI measured  but judged clinically | - | - | 1 | - | - | - | - |
| **≥ 2 underlying medical conditions** | 4/1418 (0.3%) | 6/1556 (0.4%) | 1/132* | 0/731  (0%) | 3/490  (0.6%) | 0/35* | 3/525* |
| **Other factors of concern** |  |  |  |  |  |  |  |
| Tuberculosis | Na | Na | Na | Na | Na | Na | Na |
| **Outcome** |  |  |  |  |  |  |  |
| Non-ICU | Na | Na | 14/28* | Na | Na | 15/16* | 15/16* |
| ICU | Na | Na | 14/28* | Na | Na | 1/16* | 1/16* |
| Deaths | 4/1413 (0.3%) | 4/1543 (0.3%) | 19/141* | 10/730 (13.7%) | 0/489  (0%) | 0/2* | 10/1219 (0.8%) |
| **Respiratory support** |  |  |  |  |  |  |  |
| Oxygen | - | - | 4/5* | - | - | 1/7* | 1/7* |
| Ventilation | - | - | - | - | - | 2/7* | 2/7* |
| Both O2 and ventilation | - | - | - | - | - | - | - |
| No support required | - | - | 1/5* | - | - | 4/7* | 4/7* |
| **Diagnosis pneumonia** |  |  |  |  |  |  |  |
| based on clinical symptoms | Na | Na | 24/42*  (57%) | Na | Na | 7/23* | 7/23* |
| **Influenza vaccination received** (within 14 days before symptom onset) | 3/595* | 14/1223* | 2/116* | 2/123* | 8/396* | 0/1* | Na* |
| **Antiviral treatment** received <48 hrs after onset | Na | Na | Na | Na | Na | Na | Na |

Na: Not available, * >10% incomplete data; *Note: The total number of SARI cases was 4667, but there were missing data on the age and/or season variables for 17 cases and therefore excluded from the table.*

**10. POOLED DATA ANALYSIS**

**Table 10-I Pooled data analysis -** Univariate logistic regression - Association risk factor (p-value, chi square test/Continuity correction/ Fisher’s exact test) for three severity outcomes (p-value, OR and 95% CI) for influenza-positive patients.

|  | **ICU vs non-ICU**  **and non-fatal**  **(n=914)** | **FATAL vs non-ICU**  **and non-fatal**  **(n=772)** | **ICU/FATAL vs non-ICU and non-fatal**  **(n=1046)** |
| --- | --- | --- | --- |
|  |  |  |  |
| Dummy_Albania | p=0.308 | **OR=1.78**  **(0.92-3.47)**  **p=0.126** | **OR=1.55**  **(0.95-2.52)**  **p=0.103** |
| Dummy_Georgia | **OR=1.31**  **(0.10-1.76)**  **p=0.095** | p=0.453 | **OR=1.24**  **(0.95-1.61)**  **p=0.128** |
| Age, 2cat  (0-15, 15+) | p=0.849 | **OR=10.80**  **(6.18-18.88)**  **p=0.000** | **OR=1.89**  **(1.47-2.43)**  **p=0.000** |
| Age, 4cat  (<5, 5-14, 15-49, 50+) | p=0.146 | p=0.000 | p=0.000 |
| Age, 7cat (<2, 2-5, 5-14, 14-29, 30-49, 50-64, 65+) | p=0.005 | p=0.000 | p=0.000 |
| Underlying condition  (no/yes) | OR=2.355  (1.77-3.17)  p=0.000 | OR=41.47  (20.81-84.24)  p=0.000 | OR=4.53  (3.47-5.91)  p=0.000 |
| >2 underlying conditions  (no/yes) | OR=2.04  (1.28-3.25)  p=0.004 | OR=15.91  (10.09-25.11)  p=0.000 | OR=4.91  (3.36-7.17)  p=0.000 |
| Diabetes  (no/yes) | p=0.913 | **OR=4.21**  **(2.07-8.59)**  **p=0.000** | OR=1.94  (1.03-3.66)  p=0.056 |
| Lung disease  (no/yes) | **OR=5.23**  **(3.46-7.91)**  **p=0.000** | **OR=21.60**  **(13.55-34.41)**  **p=0.000** | OR=8.681  (5.971-12.62)  p=0.000 |
| Heart disease  (no/yes) | **OR=1.99**  **(1.27-3.11)**  **p=0.003** | **OR=7.96**  **(5.07-12.49)**  **p=0.000** | **OR=3.58**  **(2.46-5.20)**  **p=0.000** |
| Kidney disease  (no/yes) | p=1.000 | **OR=11.00**  **(3.76-32.20)**  **p=0.000** | **OR=4.12**  **(1.46-11.63)**  **p=0.008** |
| Liver disease  (no/yes) | p=0.763 | **p=0.001**  **OR=5.51**  **(2.09-14.55)** | **OR=2.56**  **(1.05-6.23)**  **p=0.055** |
| Neurological disease  (no/yes) | p=0.557 | **OR=6.33**  **(1.40-28.64)**  **p=0.021** | p=0.443 |
| Pregnant  (no/yes) | **OR=2.51**  **(1.27-4.94)**  **p=0.011** | **OR=4.77**  **(2.35-9.70)**  **p=0.000** | **OR=3.07**  **(1.68-5.60)**  **p=0.000** |
| Immune (no/yes) | p=0.811 | P=0.169 | p=0.787 |
| Obesity*  (no/yes) | OR=0.49  (0.26-0.93)  p=0.038 | OR=1.77  (0.96-3.24)  p=0.091 | p=0.574 |
| Cancer (no/yes) | p=0.862 | p=0.637 | p=1.000 |
| A(H1N1)pdm09  (no/yes) | **OR=0.38**  **(0.28-0.51)**  **p=0.000** | p=0.383 | **OR=0.53**  **(0.40-0.68)**  **p=0.000** |

*The risk factor obesity was excluded from the multivariate analysis as there were a large proportion of missing data on this variable.

*Risk factors with p < 0.15, marked in bold, are included in the multivariate logistic regression analysis. There was a high correlation (>0.5) between the three age groups variables, therefore only the age group with two categories was included in the multivariate analysis. In addition the underlying condition and >2 underling conditions were correlated with individual underlying conditions and therefore we only included the individual underlying in the multivariate analysis.*

**Table 10-II Pooled data analysis -** Multivariate logistic regression.

*For the two severity levels ICU and ICU/FATAL no results are presented because the Hosmer and Lemeshow test was significant (p=0.000) indicating a bad fit with the model.*

|  | **FATAL vs non-ICU**  **and non-fatal**  **(n=772)** |
| --- | --- |
| Dummy Albania (ref cat=Romania) | p=0.900 |
| Age cat (0-14, 15+) | **OR=5.44**  **(2.77-10.71)**  **p=0.000** |
| Diabetes (no/yes) | p=0.344 |
| Lung disease  (no/yes) | **OR=14.89**  **(8.62-25.71)**  **p=0.000** |
| Liver disease  (no/yes) | **OR=3.59**  **(1.11-11.42)**  **p=0.032** |
| Heart disease  (no/yes) | **OR=4.01**  **(2.21=7.29)**  **p=0.000** |
| Kidney disease  (no/yes) | **OR=3.88**  **(1.06-14.28)**  **p=0.041** |
| Neurological disease  (no/yes) | p=0.386 |
| Pregnant  (no/yes) | **OR=7.08**  **(3.01-16.68)**  **p=0.000** |
